# Dual Effect: High NADH Levels Contribute to Efflux-Mediated Antibiotic Resistance but Drive Lethality Mediated by Reactive Oxygen Species

**DOI:** 10.1128/mbio.02434-21

**Published:** 2022-01-18

**Authors:** Alejandro Arce-Rodríguez, Debbie Pankratz, Matthias Preusse, Pablo I. Nikel, Susanne Häussler

**Affiliations:** a Department of Molecular Bacteriology, Helmholtz Centre for Infection Research, Braunschweig, Germany; b Department of Molecular Bacteriology, Twincore, Hannover, Germany; c The Novo Nordisk Foundation Center for Biosustainability, Technical University of Denmark, Kongens Lyngby, Denmark; d Department of Clinical Microbiology, Copenhagen University Hospital – Rigshospitalet, Copenhagen, Denmark; e Cluster of Excellence RESIST (EXC 2155), Hannover Medical School, Hannover, Germany; Louis Stokes Veterans Affairs Medical Center

**Keywords:** NAD(P)H, redox potential, Pseudomonas aeruginosa, NADH oxidase, formate dehydrogenase, antibiotic resistance, efflux pumps, membrane potential, metabolism, NAD(P)H

## Abstract

In light of the antibiotic crisis, emerging strategies to sensitize bacteria to available antibiotics should be explored. Several studies on the mechanisms of killing suggest that bactericidal antibiotic activity is enforced through the generation of reactive oxygen species (ROS-lethality hypothesis). Here, we artificially manipulated the redox homeostasis of the model opportunistic pathogen Pseudomonas aeruginosa using specific enzymes that catalyze either the formation or oxidation of NADH. Increased NADH levels led to the activation of antibiotic efflux pumps and high levels of antibiotic resistance. However, higher NADH levels also resulted in increased intracellular ROS and amplified antibiotic killing. Our results demonstrate that growth inhibition and killing activity are mediated via different mechanisms. Furthermore, the profound changes in bioenergetics produced low-virulence phenotypes characterized by reduced interbacterial signaling controlled pathogenicity traits. Our results pave the way for a more effective infection resolution and add an antivirulence strategy to maximize chances to combat devastating P. aeruginosa infections while reducing the overall use of antibiotics.

## INTRODUCTION

In just over 100 years, antibiotics have changed the world of medicine. They are widely used and have been instrumental in the treatment and prevention of infectious diseases. However, today, bacterial resistance to commonly used antibiotics continues to grow, and the frequency and range of infections that cannot be treated with available antimicrobial therapies have increased at an alarming rate worldwide (1, 2). Consequently, there is a clear and growing need for new antibiotics. However, particularly for the treatment of infections caused by antibiotic-resistant Gram-negative bacterial pathogens, there are almost no compounds in the clinical development pipeline (3). One particularly problematic opportunistic pathogen is Pseudomonas aeruginosa. This bacterium plays a dominant role as a causative agent of severe nosocomial infections, and it also determines morbidity and mortality in chronically infected cystic fibrosis patients (4). The decreasing number of antibiotic options against multidrug-resistant Gram-negative pathogens underlines the need to optimize current therapies. In addition to an intensified search for novel antimicrobials, one might explore possibilities to sensitize bacteria to currently available antibiotics. This could lead to a more effective killing of the infecting bacteria and reduce the total amount of antibiotics used. Both a more effective killing and a reduction in overall antibiotic consumption promise to mitigate selection pressure for resistance and thus slow down resistance development. For this approach to be successful, however, requires not only detailed knowledge of the resistance mechanism but also more insight into the mechanisms of killing.

In 2007, J. J. Collins research group provided new evidence that the activity of bactericidal antibiotics is linked to reactive oxygen species (ROS) production (5). They hypothesized that, in addition to the drug-specific inhibition of cellular targets, antibiotic activity drives ROS production, which contributes in a general way to lethal antibiotic activity. It was initially postulated that altered energy metabolism following antibiotic exposure was the source of ROS (5, 6). It was also hypothesized that the bactericidal antibiotic activity stimulates ATP-dependent repair processes and that the sudden increased demand for ATP stimulates metabolic action (7). Consequently, a burst of respiration produces elevated levels of ROS as by-products of aerobic respiration when the electron transfer chain exceeds its normal capacity.

Any disturbance of the intracellular redox potential can lead to increased ROS production and thus critically affect cellular health and function. The redox potential, which is determined by the balance of oxidants and reductants in the cell but also by pH, is therefore tightly regulated (8). Antibiotic-induced hyperrespiration might not only lead to the hypergeneration of ROS but also to a drain of protons from the cytoplasm and—if not replaced through proton reentry via ATP synthesis—will also affect pH homeostasis. In fact, it was suggested that the effects of antibiotics on pH homeostasis should be considered a potential mechanism that contributes to antibiotic lethality (9, 10). As the chemistries of protons and electrons are closely linked, it may be not possible to decipher whether the bactericidal effect of an antibiotic is due to oxidative stress, the disruption of pH homeostasis, or both.

In this study, we aimed at shedding more light on the assumption that disturbances of the redox balance causally contribute to cell death and is not simply associated with it. Antioxidants and iron chelators exhibiting ROS scavenging activities have been demonstrated to protect cells from antibiotic killing (5, 11, 12). Here, we took the opposite approach and by means of actively manipulating the NADH-to-NAD^+^ ratio increased ROS production. We investigated whether a shift in the redox balance influences the bactericidal activity of antibiotics and thus supports the hypothesis that, in addition to inducing lethal cellular damage via interference with target-specific processes, antibiotics disturb the redox balance that thus induce an additional component of toxicity, which contributes causally to drug lethality.

## RESULTS

### Redox cofactor engineering in P. aeruginosa.

**FIG 1:**
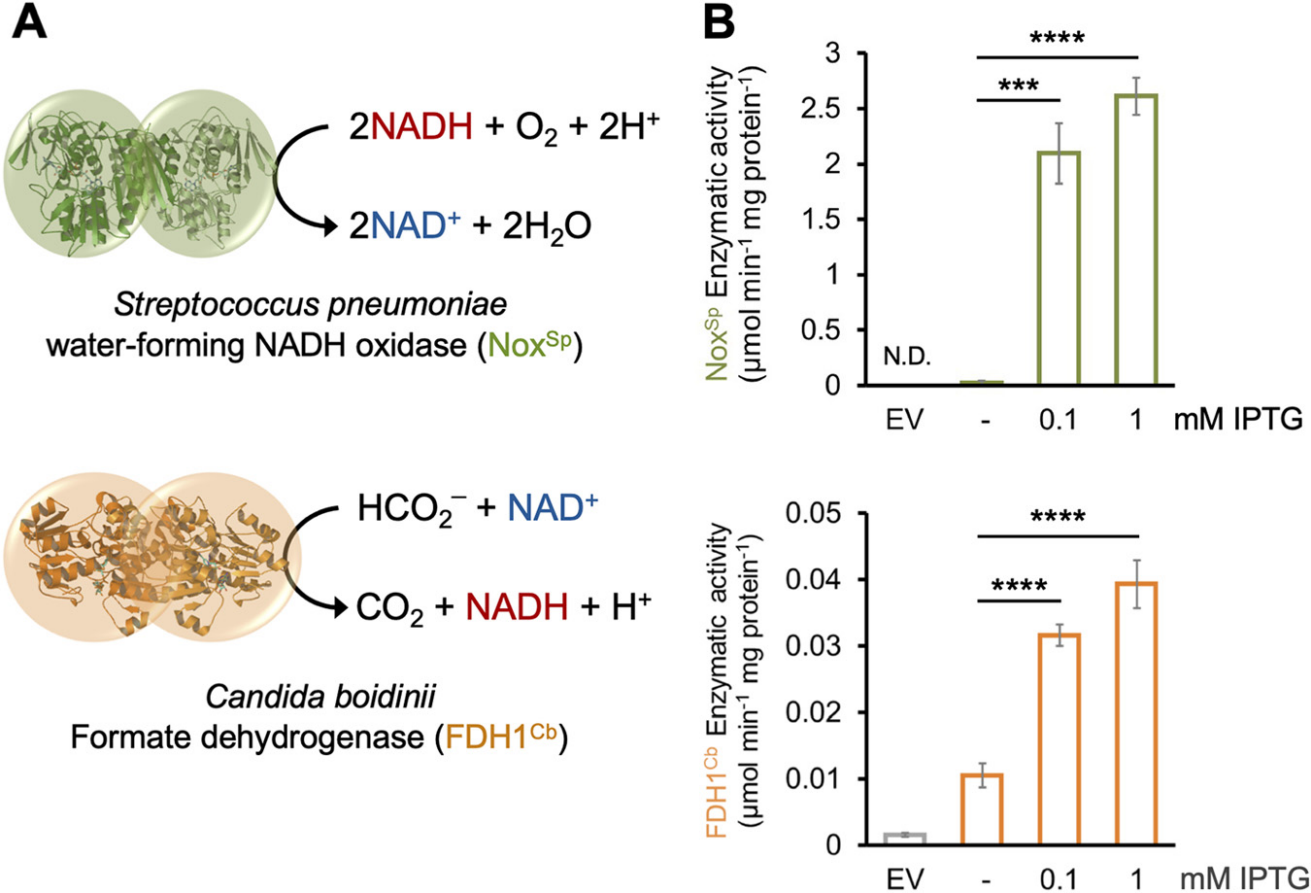
Manipulation of the NADH-to-NAD^+^ ratio via the introduction of an NADH oxidase and a formate dehydrogenase into PA14. (A) Enzymatic reactions of the S. pneumoniae NADH oxygenase Nox^Sp^ and the C. boidinii formate dehydrogenase *FDH1^Cb^*. (B) Enzymatic activity (in μmol per min and mg total protein) of both enzymes increased upon addition of increasing concentrations of β-d-1-thiogalactopyranoside (IPTG). The activities were measured in cell extracts. EV stands for cells carrying the empty vector. Significant differences were calculated by unpaired *t* test with Welch’s correction and are indicated with asterisks. ***, *P* < 0.001; ****, *P* < 0.0001. N.D., not determined.

The reducing equivalents NADH and NADPH act as carriers of electrons generated from substrate oxidation and serve as important cofactors in diverse redox reactions. Not only does the total NAD(P)H pool determine the reaction activity of the redox reactions within the cell, but the speed of the reaction is also influenced by the ratio of the reduced to the oxidized form of these molecules. Several enzymatic methods have been developed for the regeneration of NAD(P)H (13). By overexpressing the NAD^+^-dependent formate dehydrogenase (FDH) from the yeast Candida boidinii in Escherichia coli, the maximum yield of NADH was shown to be doubled from 2 to 4 mol NADH/mol glucose consumed (14). In this study, we increased the NADH-to-NAD^+^ ratio via the expression of the C. boidinii FDH (*FDH1*^Cb^) in P. aeruginosa grown in the presence of formate and thus catalyzed the transformation of formate to CO_2_ while simultaneously reducing NAD^+^ to NADH (14). We also introduced the *nox* gene from Streptococcus pneumoniae (*nox*^Sp^) (15) into P. aeruginosa. This gene encodes a NADH-specific oxidase (Nox), which catalyzes the reduction of O_2_ to H_2_O together with a negligible formation of H_2_O_2_ (Fig. 1A). Thus, the Nox^Sp^-mediated reduction of NADH is accomplished without a significant contribution to the formation of ROS (16). Both genes were cloned under the control of an isopropyl β-d-1-thiogalactopyranoside (IPTG)-inducible expression system and were mobilized into P. aeruginosa PA14.

A boost in the activity of both enzymes was observed in cell extracts obtained from cells harvested after addition of increasing IPTG concentrations, indicating that P. aeruginosa cells produce fully functional enzymes (Fig. 1B and C). We also observed that the redox imbalance generated by overexpressing either of the two enzymes caused a slight decrease in growth rate and a prolonged lag phase compared to the cells carrying the empty plasmid (Fig. S1).

10.1128/mBio.02434-21.3 FIG S1 Growth parameters of *P. aeruginosa* PA14 overexpressing (A) *nox*^Sp^ and (B) *FDH1*^Cb^ strains. Download FIG S1, PDF file, 0.2 MB. Copyright © 2022 Arce-Rodríguez et al. 2022 Arce-Rodríguez et al. https://creativecommons.org/licenses/by/4.0/ This content is distributed under the terms of the Creative Commons Attribution 4.0 International license.

**FIG 2:**
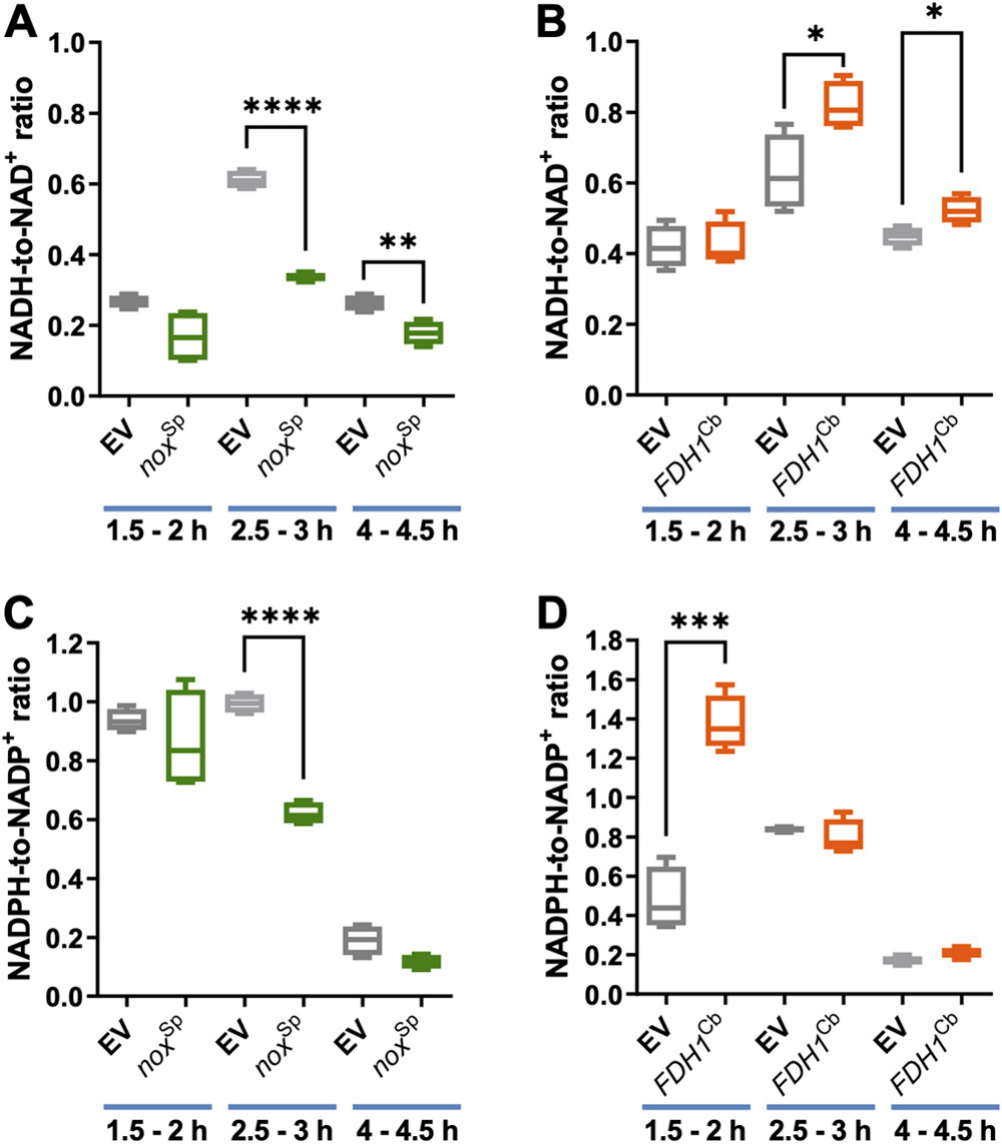
Quantification of the NAD(P)H/NAD(P)^+^ ratios in P. aeruginosa overexpressing *nox*^Sp^ and *FDH1*^Cb^. The NADH-to-NAD^+^ ratio (A, B) and the NADPH-to-NADP^+^ ratio (C, D) were determined by a nucleotide cycling assay in PA14 cells overexpressing either the *nox^sp^* (A, C) or *FDH1*^cb^ (B, D). PA14 harboring the empty vector (EV) served as a control. The ratios were determined following growth of the cells at different time points after IPTG induction (indicated in each case). Results of at least two independent experiments (each consisting of two technical replicates) are presented as a box plot defined by the 10th and 90th percentile. A line is drawn inside the box at the median (50th percentile). Significant differences were calculated by unpaired *t* test with Welch’s correction. *, *P* < 0.05; **, *P* < 0.01; ***, *P* < 0.001; ****, *P* < 0.0001.

We next quantified the NADH/NAD^+^ (Fig. 2A), as well as the NADPH/NADP^+^ ratios (Fig. 2B), in P. aeruginosa cells overexpressing the two enzymes at different time points following the addition of 1 mM IPTG. We observed a clear reduction of the intracellular NADH concentrations upon *nox*^Sp^ expression, while expression of *FDH1*^Cb^ caused a clear induction. This became most evident 2.5 h after IPTG induction for both strains.

Induction of *nox*^Sp^ expression also caused a clear reduction in the intracellular concentration of NADPH, while a very strong increase was observed for NADPH in *FDH1*^Cb^ induced cells. Thereby, it seemed that the effect of FDH1^Cb^ already became apparent after an induction with IPTG for less than 2 h.

### High intracellular NADH levels induce the formation of ROS and increase the membrane potential.

**FIG 3:**
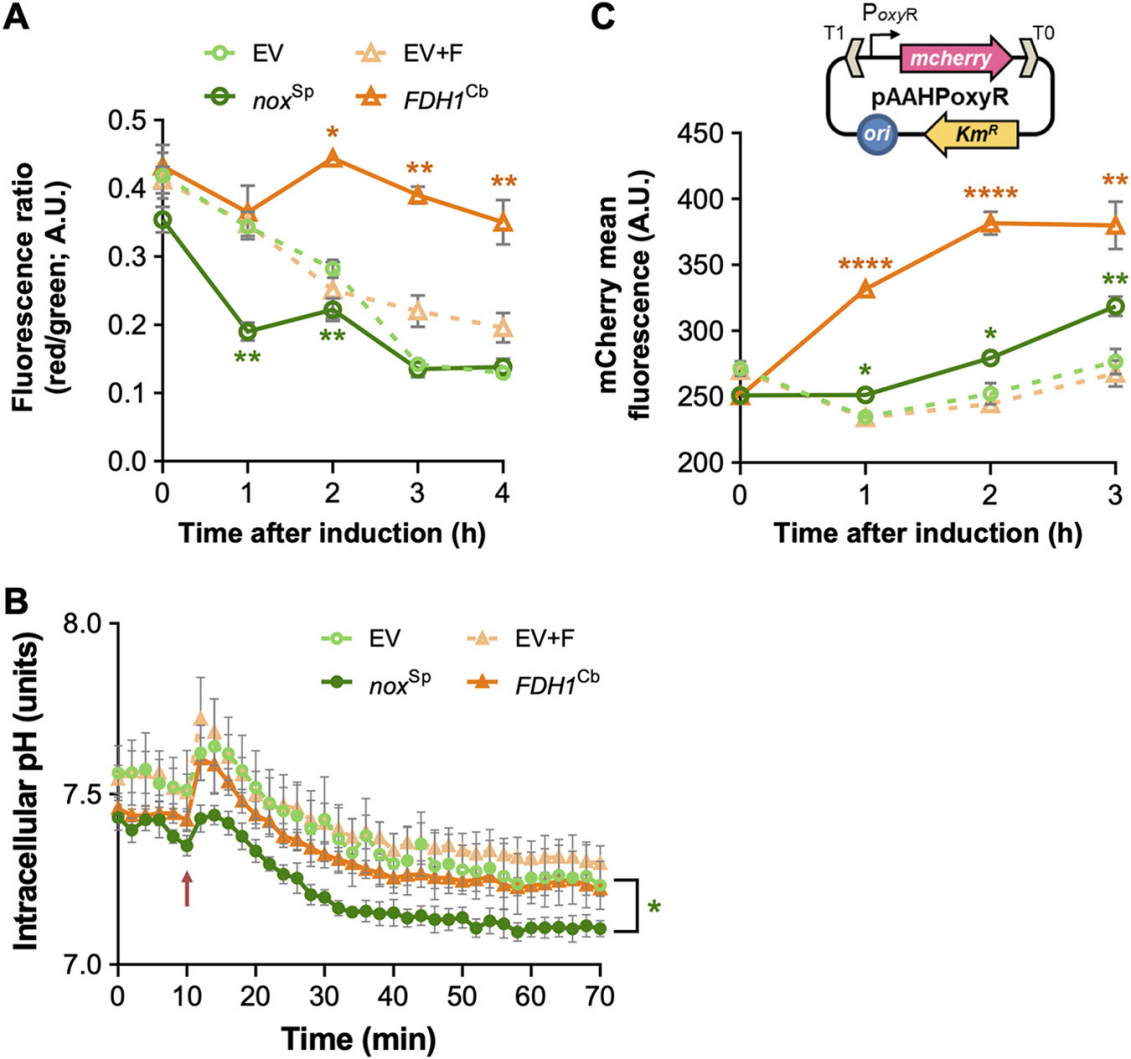
Membrane potential, intracellular pH, and ROS formation in P. aeruginosa overexpressing *nox*^Sp^ and *FDH1*^Cb^ strains. (A) The membrane potential was determined by means of the fluorescent membrane potential indicator dye DiOC_2_ in PA14 overexpressing either *nox*^Sp^ or *FDH1*^Cb^. Cells carrying the empty vector (EV) served as a control. Induction of the cultures was achieved in the early exponential phase of growth using 1 mM IPTG. A reduction in the fluorescence ratio (red/green) indicates a decrease in the membrane potential. The experiment was carried out in triplicate. (B) The intracellular pH was calculated using the ratiometric indicator protein PHP encoded in plasmid pS2513·PHP. Cells harboring the pH sensor and the plasmids encoding *nox*^Sp^ or *FDH1*^Cb^ were grown in microtiter plates, and the pH was determined by calculating the ratio between the fluorescence peaks at λ_excitation_ = 405 nm and λ_excitation_ = 485 nm, as described in the experimental procedures. The time of induction with 1 mM IPTG is depicted in the plot with a red arrow. The average results of at least three independent replicates with their respective standard deviations are shown. (C) The formation of ROS was recorded in cells carrying the pAAHPoxyR reporter plasmid (caption in Fig. 3C). This Km-resistant vector couples the transcriptional activation of the *mcherry* fluorescent protein with the ROS-responsive *oxy*R promoter (P*_oxy_*_R_). Cells carrying this ROS sensor and the plasmids expressing either *nox*^Sp^ or *FDH1*^Cb^ were grown to the early exponential phase of growth and induced with 1 mM IPTG. The mCherry-specific signal was recorded in a flow cytometer, and the mean fluorescence intensities were calculated from the whole-cell population. The symbols for each sample within the plot are the same as in Fig. 3A The experiment was carried out in triplicate. Significant differences in panels A and C were determined by unpaired *t* test with Welch’s correction: *, *P* < 0.05; **, *P* < 0.001; ****, *P* < 0.0001. In the case of the pH measurements (B), a Welch’s two-sample *t* test on ranked data was performed. The *P* values for the different time points were corrected using the false discovery rate (FDR). Check the experimental procedures and Fig. S6 for details.

In *FDH1*^Cb^-expressing P. aeruginosa, the sudden increase of NADH generates a disproportion in the redox equivalents of the cell. The cell can counteract this metabolic imbalance by an increased electron transport chain (ETC) activity, which will regenerate NAD^+^ and thus reestablish the redox homoeostasis. As a result of the higher respiratory rates, the inner membrane should become more hyperpolarized. Indeed, staining the *FDH1*^Cb^-overexpressing cells with the fluorescent membrane potential indicator dye DiOC_2_ revealed an increased membrane potential for prolonged time periods (Fig. 3A), while *nox*^Sp^-positive cells exhibited a reduced membrane potential. In addition, hyperactivation of respiration is expected to alter the intracellular pH due to a drain of intracellular protons (17). Indeed, Fig. 3B shows a significant drop of the pH value as determined by the pH ratiometric protein PHP upon overexpression of *nox*^Sp^ as opposed to *FDH1*^Cb^-overexpressing cells.

10.1128/mBio.02434-21.8 FIG S6 Statistical differences of intracellular pH values. Download FIG S6, PDF file, 0.2 MB. Copyright © 2022 Arce-Rodríguez et al. 2022 Arce-Rodríguez et al. https://creativecommons.org/licenses/by/4.0/ This content is distributed under the terms of the Creative Commons Attribution 4.0 International license.

Hyperactivation of respiration has been previously demonstrated to stimulate the formation of ROS when the electron transfer chain exceeds its normal capacity (5, 18). In order to measure oxidative stress levels in the cells, we constructed a plasmid-borne, oxidative stress transcriptional reporter and measured the promoter activity of *oxyR* following its fusion to the gene encoding mCherry (Fig. 3C). The *oxyR* promoter activity is widely used as readout to measure oxidative stress (11, 19). The transcriptional regulator OxyR becomes activated by H_2_O_2_ to promote the expression of many ROS-response genes, such as *katA*, *katB*, *ahpB*, and *ahpCF* (20). Exposing P. aeruginosa cells transformed with the plasmid reporter to conditions previously known to generate ROS (such as treatment with H_2_O_2_, methyl viologen or antibiotics) led to significant increases in mCherry fluorescence (Fig. S2). We next monitored the redox reporter activity in P. aeruginosa carrying both the *oxyR* reporter system and the plasmids expressing either *nox*^Sp^ or *FDH1*^Cb^ (or the empty plasmid controls; Fig. 3C). The presence of *nox*^Sp^ produced only a slight increase in the oxidative stress levels. In addition to their water-forming nature, NADH oxidases produce minor H_2_O_2_ concentrations if molecular oxygen (O_2_) is not fully reduced to H_2_O (21, 22). Thus, the small increase in ROS observed in the *nox*^Sp^ strain could be caused by its strong induction as it has been previously observed for the homologous enzymes from Streptococcus mutans (23) or Lactobacillus pentosus (24). The overexpression of *FDH1*^Cb^ led to a clear enhancement of the *oxyR* promoter activity (i.e., increase in ROS formation). In summary, it appears that an increase in cellular NADH levels following overexpression of *FDH1*^Cb^ results in the generation of ROS as a consequence of electron transport chain activation to balance the NAD^+^/NADH redox potential. Hyperrespiration is associated with a loss of intracellular protons in *FDH1*^Cb^-overexpressing cells, which is reflected in an increased membrane potential, while in *nox*^Sp^-overexpressing cells, we observe a reduction in the membrane potential and a significant decrease in intracellular pH.

10.1128/mBio.02434-21.4 FIG S2 Evaluation of the pAAHPoxyR2 as an intracellular ROS reporter. Download FIG S2, PDF file, 0.1 MB. Copyright © 2022 Arce-Rodríguez et al. 2022 Arce-Rodríguez et al. https://creativecommons.org/licenses/by/4.0/ This content is distributed under the terms of the Creative Commons Attribution 4.0 International license.

### The redox potential is linked to antimicrobial resistance in P. aeruginosa.

**FIG 4:**
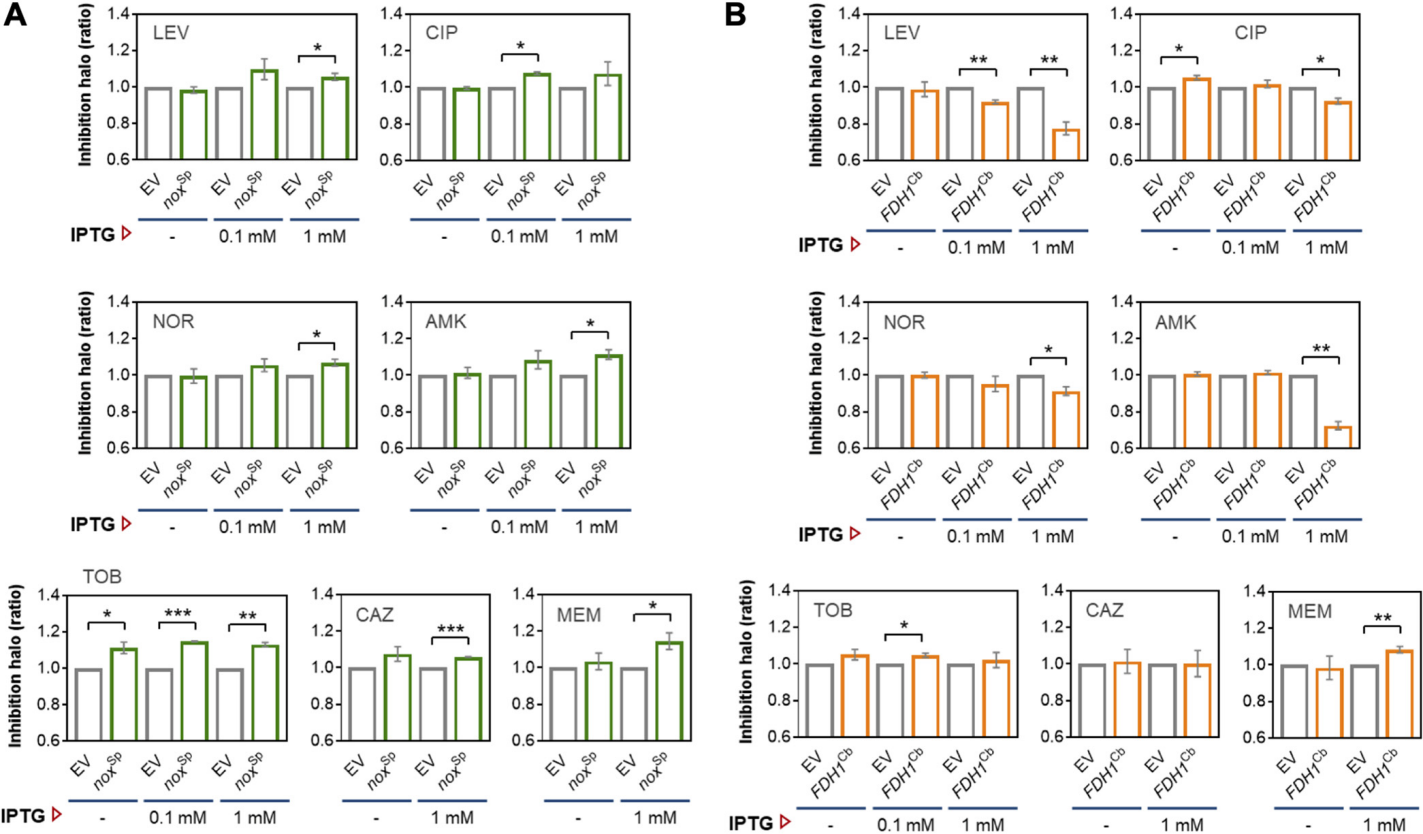
Antimicrobial disk diffusion assay in P. aeruginosa overexpressing *nox*^Sp^ and *FDH1*^Cb^. PA14 cells expressing *nox*^Sp^ (A) or *FDH1*^Cb^ (B) were plated onto LB-agar with or without the inducer IPTG (0.1 sor 1 mM as indicated in each case) and challenged to each antibiotic-impregnated disk. Cells carrying the empty vector (EV) served as a control, and 10 mM sodium formate was supplemented to the agar plates used for the *FDH1*^Cb^ strain and the respective vector control. The diameter of the inhibition zone of at least three independent experiments were measured and normalized to the empty vector control in each case, as depicted. Significant differences between samples were calculated by means of a ratio paired *t* test and are indicated by asterisks. *, *P* < 0.05; **, *P* < 0.01; ***, *P* < 0.001. LEV, levofloxacin; CIP, ciprofloxacin; NOR, norfloxacin; AMK, amikacin; TOB, tobramycin; CAZ, ceftazidime; MEM, meropenem.

Previous studies indicate that oxidative stress is involved in antibiotic killing of several bacterial species (5, 6, 11), and conversely it seems conceivable that oxidative conditions also contribute to antibiotic resistance. We therefore performed a disk diffusion antimicrobial susceptibility test and recorded the antibiotic resistance profiles of P. aeruginosa cells carrying either of the two enzymes (*nox*^Sp^ or *FDH1*^Cb^) or the empty vector for several antibiotics belonging to the different classes of the fluoroquinolones (norfloxacin, levofloxacin, ciprofloxacin), aminoglycosides (tobramycin, amikacin), and β-lactams (ceftazidime, meropenem). The strain overexpressing *nox*^Sp^ exhibited increased sensitivity to all tested antibiotics, characterized by a slightly larger inhibition zones (on average 0.1-fold) compared to the cells with the empty vector (Fig. 4A). In contrast, the cells overexpressing *FDH1*^Cb^ showed increased resistance to fluoroquinolones as observed with reduced inhibition zones, especially levofloxacin (0.25-fold) and to a lesser extent for ciprofloxacin and norfloxacin (both showing an ∼0.1-fold reduction), as well as for the aminoglycoside amikacin (0.27-fold), but not tobramycin or the β-lactams meropenem and ceftazidime (Fig. 4B). In *FDH1*^Cb^-induced cells, we also observed a growth advantage in liquid cultures supplemented with increasing amounts of amikacin and the quinolones compared to the empty vector control (Fig. S3). To further confirm these results, we repeated the antimicrobial susceptibility testing by means of an Etest. P. aeruginosa cells that overproduced NADH via expression of *FDH1*^Cb^ acquired a 4-fold increased resistance toward quinolones (levofloxacin and ciprofloxacin) and a 3-fold increased resistance toward amikacin (Table 1). Noticeably, upon induction of *FDH1*^Cb^, we observed a >2-fold reduction in the minimal inhibitory concentration (MIC) values for ceftazidime and meropenem, which was in the same order of magnitude as we observed when testing the *nox*^Sp^-positive P. aeruginosa strain. This suggests that expression of either of the two enzymes generates a metabolic imbalance that impairs cell homoeostasis and thus may generally enhance susceptibility of the cells toward β-lactam antibiotics. Nevertheless, a disturbed redox balance characterized by the accumulation of NADH enhanced antibiotic resistance toward antibiotics belonging to the antibiotic classes of aminoglycosides (amikacin) and fluoroquinolones (norfloxacin, levofloxacin, and ciprofloxacin), but not to the β-lactams (meropenem and ceftazidime).

**TABLE 1:** Antimicrobial susceptibility in P. aeruginosa PA14 overexpressing *nox*^Sp^ or *FDH1*^Cb^ as determined by the Etest^*a*^

Antimicrobial	MIC (μg mL^−1^)^*b*^							
	Empty vector		*nox* ^Sp^		Empty vector^*c*^		*FDH1*^Cb^ ^*c*^	
	Not induced	1 mM IPTG	Not induced	1 mM IPTG	Not induced	1 mM IPTG	Not induced	1 mM IPTG
Levofloxacin	0.38	0.38	0.25	0.25	0.38	0.38	0.38	1.5
Ciprofloxacin	0.125	0.19	0.125	0.094	0.125	0.19	0.125	0.5
Amikacin	3	4	3	2	3	4	3	12
Tobramycin	2	4	1	1.5	2	3	1	2
Meropenem	0.25	0.25	0.25	0.125	0.25	0.25	0.25	0.094
Ceftazidime	0.5	0.75	0.38	0.38	0.5	0.5	0.5	0.25

a MIC, minimal inhibitory concn; IPTG, isopropyl β-d-1-thiogalactopyranoside.

b Reported by the Etest assay.

c LB-agar supplemented with 10 mM sodium formate.

10.1128/mBio.02434-21.5 FIG S3 Growth curves of *nox*^Sp^- and *FDH1*^Cb^-overexpressing *P. aeruginosa* PA14 challenged with different classes of antibiotics. Download FIG S3, PDF file, 1.1 MB. Copyright © 2022 Arce-Rodríguez et al. 2022 Arce-Rodríguez et al. https://creativecommons.org/licenses/by/4.0/ This content is distributed under the terms of the Creative Commons Attribution 4.0 International license.

### Increased NADH levels produced by *FDH1*^Cb^ confer an increased MICs by facilitating transport across MexAB and MexXY efflux pumps.

Resistance typically results from antibiotic target alteration (e.g., in the quinolone resistance determining regions) or enzymatic drug inactivation (e.g., by plasmid- or chromosome-encoded aminoglycoside modifying enzymes). Nevertheless, resistance elicited by reduced uptake or increased efflux of antibiotics is also commonplace. The P. aeruginosa genome encodes four intrinsic, highly efficient resistance nodulation division (RND) family multidrug efflux pumps, namely, MexAB-OprM, MexCD-OprJ, MexEF-OprN, and MexXY-OprM. The RND efflux pumps operate as drug/proton antiporters, and their activity has been shown to lead to intracellular H^+^ accumulation (25, 26). We hypothesized that the hyperpolarization of the cell membrane caused by the excess of NADH could influence the activity of the drug antiporters. We therefore introduced the plasmids carrying either of the two enzymes (*FDH1*^Cb^ or *nox*^Sp^), as well as the empty vector control into individual multidrug efflux-pump inactivated PA14 mutants Δ*mex*AB, Δ*mex*CD, Δ*mex*EF, and Δ*mex*XY; (27). We then reanalyzed the antimicrobial susceptibility by disk diffusion for each of the mutant strains and determined the ratio between the diameter of the inhibition zone of the *nox*^Sp^ and the *FDH1*^Cb^ strain versus the empty plasmid controls. As observed before in the PA14 wild type, induction of *nox*^Sp^ in all four efflux pump mutants produced slightly larger zones of inhibition on the agar plates and thus led to increased susceptibility against all tested antibiotics (Fig. S4).

10.1128/mBio.02434-21.6 FIG S4 Antimicrobial disk diffusion assay in *P. aeruginosa* efflux pump mutants overexpressing *nox*^Sp^. Download FIG S4, PDF file, 0.7 MB. Copyright © 2022 Arce-Rodríguez et al. 2022 Arce-Rodríguez et al. https://creativecommons.org/licenses/by/4.0/ This content is distributed under the terms of the Creative Commons Attribution 4.0 International license.

**FIG 5:**
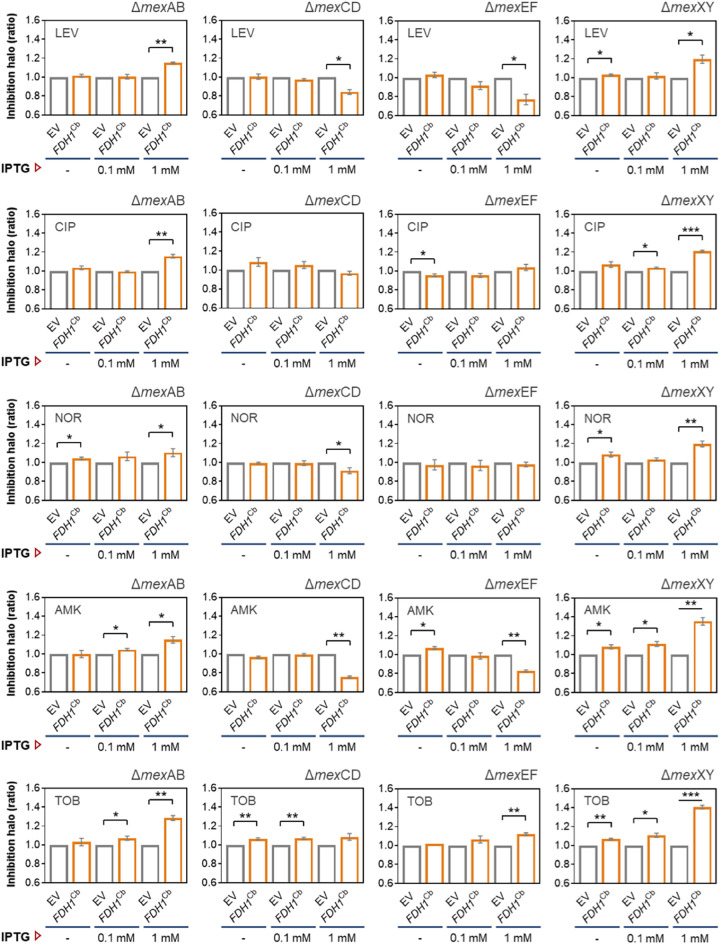
Antimicrobial disk diffusion assay in P. aeruginosa efflux pump mutants overexpressing *FDH1*^Cb^. Deficient strains in the main RND efflux pumps (Δ*mex*AB, Δ*mex*CD, Δ*mex*EF, and Δ*mex*XY) expressing *FDH1*^Cb^ were plated onto LB-agar with or without the inducer IPTG (0.1 mM or 1 mM, as indicated) and challenged with antibiotic-impregnated disks. Cells carrying the empty vector (EV) served as a control, and 10 mM sodium formate were supplemented to the agar plates used for the *FDH1*^Cb^ strain and the respective vector control. The diameter of the inhibition zone of at least three independent experiments were measured and normalized to the empty vector control as described in Fig. 4. Significant differences between samples were calculated by means of a ratio paired *t* test and are indicated by asterisks. *, *P* < 0.05; **, *P* < 0.01; ***, *P* < 0.001. LEV, levofloxacin; CIP, ciprofloxacin; NOR, norfloxacin; AMK, amikacin; TOB, tobramycin.

When *FDH1*^Cb^ was induced in both the *mexCD* and *mexEF* deletion mutants, the cells exhibited similar resistance patterns as observed before for the PA14 wild type: *FDH1*^Cb^ overexpression leads to increased resistance levels against levofloxacin and amikacin (Fig. 5). However, this resistance phenotype was lost if *FDH1*^Cb^ was overexpressed in the *mexAB* or *mexXY* deletion mutants. These results indicate that the activity of the MexAB and MexXY efflux pumps contribute to the aminoglycoside and fluoroquinolone resistance phenotype of the *FDH1*^Cb^ strain. Not only did inactivating *mex*AB and *mex*XY abolish the observed levofloxacin and amikacin resistance in PA14 *FDH1*^Cb^-overexpressing strains, but their inactivation also led to an even higher sensitivity against all the antibiotics (levofloxacin, norfloxacin, amikacin, and tobramycin) tested in the *FDH1*^Cb^-overexpressing strains compared to the wild type (Fig. 5).

### Increased NADH levels produced by *FDH1*^Cb^ contribute to the bactericidal activity of antibiotics.

**FIG 6:**
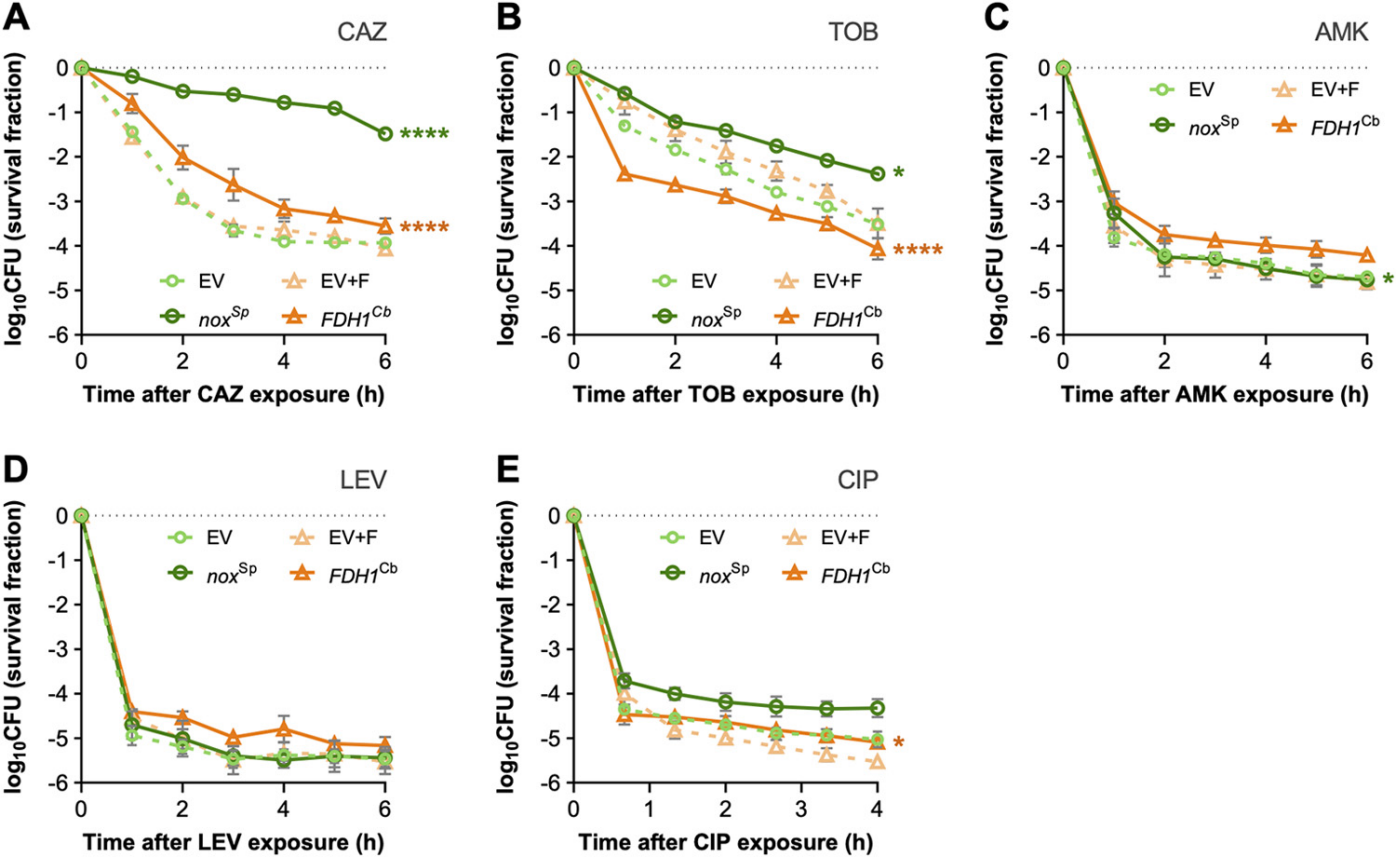
Killing curve assay in P. aeruginosa overexpressing either *nox*^Sp^ or *FDH1*^Cb^. Cells carrying the empty vector (EV) served as a control. The expression of each gene was induced with 0.1 mM IPTG, and the strains were grown until early exponential phase before the treatment with lethal concentrations of ceftazidime (A), tobramycin (B), amikacin (C), levofloxacin (D), or ciprofloxacin (E). Colony-forming units (CFU) counts were obtained from platting the cells at specific time points after antibiotic challenge and plotted as the log_10_ of the survival fraction. The average result of three independent experiments with their respective standard deviation is shown. CAZ, ceftazidime; TOB, tobramycin; AMK, amikacin; LEV, levofloxacin; CIP, ciprofloxacin. Statistical differences were calculated by the extra sum-of-squares *F* test from best-fit killing rate constants obtained by fitting the curves to nonlinear regression models, as explained in the methods. *, *P* < 0.05; ****, *P* < 0.0001.

According to the ROS hypothesis, antibiotic killing is enhanced when ROS levels increase. Thus, enhanced resistance upon overexpression of *FDH1*^Cb^, which increases oxidative stress within the cells, may appear contradictory. However, agar diffusion antimicrobial susceptibility testing is a readout for growth inhibition in the presence of the antibiotic, whereas killing is measured as bacterial survival after antibiotic exposure. We therefore induced P. aeruginosa cells with 0.1 mM IPTG (in order to minimize the effect of protein overexpression on cell growth) and exposed the cells producing either of the two enzymes (FDH1^Cb^ or Nox^Sp^) to high antibiotic concentrations (>10 times the MIC). We then plated the cells in order to determine the fraction of surviving cells over time (Fig. 6). Exposure of the cells to ceftazidime at high concentrations uncovered a significantly reduced killing of the *nox*^Sp^ bearing cells. After 6 h, the fraction of survivors was more than 250-fold higher in the *nox*^Sp^-positive cells compared to the cells bearing the empty vector. A similar trend was observed for tobramycin. Here, killing in the first hour was especially high in the *FDH1*^Cb^ carrying strain, possibly also due to higher membrane potential and thus higher aminoglycoside uptake. After 6 h the fraction of survivors was ∼15-fold higher in the *nox*^Sp^-positive cells. High antibiotic concentrations of levofloxacin, ciprofloxacin, and amikacin killed all the strains very effectively, so that no big differences between the *nox*^Sp^- or *FDH1*^Cb^-positive cells could be observed. In summary, our data are consistent with the ROS-lethality hypothesis, which states that primary antibiotic damage stimulates the accumulation of ROS, although it is not excluded that severe antibiotic damage may also lead directly to cell death (28).

### Overexpression of *FDH1*^Cb^ and *nox*^Sp^ elicit antagonistic changes in the transcriptomic profile of P. aeruginosa.

**FIG 7:**
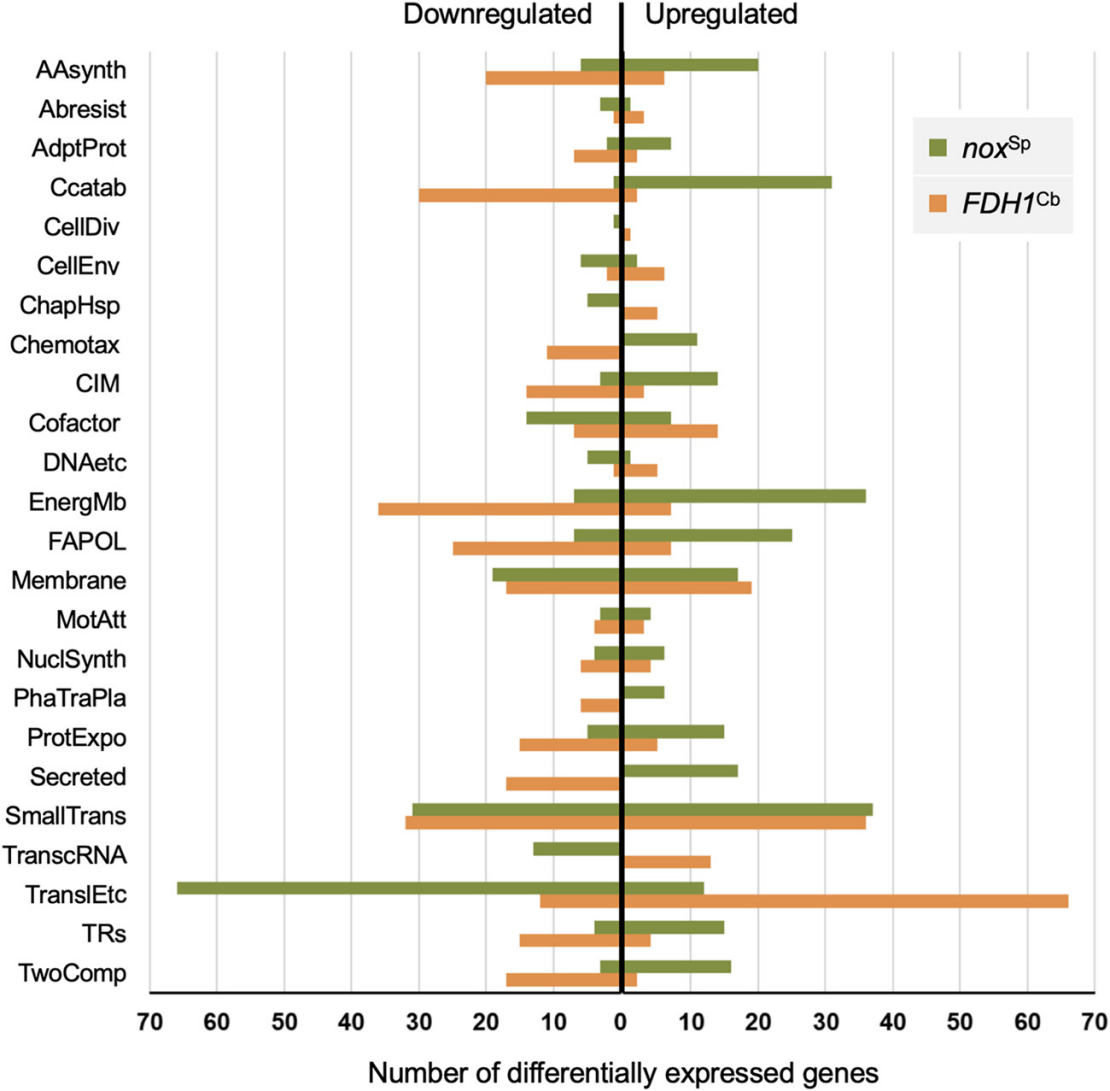
Overexpression of *nox*^Sp^ and *FDH1*^Cb^ generate antagonistic transcriptional profiles in P. aeruginosa. The 794 common genes that were found differentially regulated in both *nox*^Sp^- and *FDH1*^Cb^-producing strains are classified according to the PseudoCAP functional categories (62) and grouped according to the direction of the regulation respective to their empty vector controls (up- or downregulated). The PseudoCAP categories are abbreviated as follows: AAsynth, amino acid biosynthesis and metabolism; ABresist, antibiotic resistance and susceptibility; AdptProt, adaptation and protection; CCatab, carbon compound catabolism; CellDiv, cell division; CellEnv, Cell wall/lipopolysaccharide (LPS)/capsule; ChapHsp, chaperones and heat shock proteins; Chemotax, chemotaxis; CIM, central intermediary metabolism; Cofactor, biosynthesis of cofactors, prosthetic groups, and carriers; DNAetc, DNA replication, recombination, modification and repair; EnergMb, energy metabolism; FAPOL, fatty acid and phospholipid metabolism; Membrane, membrane proteins; MotAtt, motility and attachment; NuclSynth, nucleotide biosynthesis and metabolism; PhaTraPla, related to phage, transposon, or plasmid; ProtExpo, protein secretion/export apparatus; Secreted, secreted factors (toxins, enzymes, alginate); SmallTrans, transport of small molecules; TranscRNA, transcription, RNA processing, and degradation; TranslEtc, translation, posttranslational modification, degradation; TRs, transcriptional regulators; TwoComp, two-component regulatory systems. Hypothetical and putative genes are excluded from the figure.

We recorded the transcriptional profile of the *nox*^Sp^ and the *FDH1*^Cb^ strain versus the empty vector control in order to evaluate the global impact of changes in the redox balance on gene transcription (Table S2). Among the 968 and 2,229 genes that were differentially regulated in the *nox*^Sp^ and the *FDH1*^Cb^ strain, respectively, we found a very large proportion of 794 genes that were upregulated in the one strain and downregulated in the other (Fig. 7 and Table S2). As might have been expected, many genes involved in energy metabolism (including enzymes that shift the NADH/NAD^+^ ratio from the oxidized to the reduced form) were among those genes that were downregulated in the *FDH1*^Cb^ strain and upregulated in the *nox*^Sp^ strain. These included genes involved in carbon catabolism, structural components of the ETC cytochrome *c* oxidase (along with other cytochromes), genes encoding tricarboxylic acid (TCA) cycle components (*acn*A, *lpd*V, *fum*A, and *fum*C1) and also genes involved in the transport and degradation of amino acids and fatty acids. Genes involved in nucleic acid metabolism or the production of ribosomal and lipoproteins also exhibited a different direction of regulation (Fig. 7). Their upregulation in the *FDH1*^Cb^ strain might reflect its anabolic, growth-promoting state, while the *nox*^Sp^ strain experiences a catabolic, growth-suppressing state. Furthermore, in accordance with the assumption that the metabolic imbalance of increased NADH levels caused by the activity of *FDH1*^Cb^ is counteracted by a higher activity of the drug/proton antiporter efflux pumps, we found increased expression levels of the genes encoding several MDR efflux pumps in the *FDH1*^Cb^ strain (*mex*EF, *mex*XY, *opr*N, *ygd*E, PA14_45890, and PA14_45910).

10.1128/mBio.02434-21.2 TABLE S2 Differential gene expression between *P. aeruginosa* PA14 overexpressing *nox*^Sp^ and *FDH1*^Cb^ versus their respective empty vector controls. Download Table S2, XLSX file, 1.3 MB. Copyright © 2022 Arce-Rodríguez et al. 2022 Arce-Rodríguez et al. https://creativecommons.org/licenses/by/4.0/ This content is distributed under the terms of the Creative Commons Attribution 4.0 International license.

Interestingly, previous studies demonstrated an important role for electron transfer activity for the activation of virulence mechanisms (29). In line with this, we found that the shift to the reduced NADH form in the *FDH1*^Cb^ strain was associated with a downregulation of a set of different virulence genes, while virulence-associated genes were upregulated in cells with low NADH levels. Closer inspection of the differentially regulated genes revealed that the low-virulence gene expressing the *FDH1*^Cb^ strain exhibited reduced levels of genes involved in the production of the Pseudomonas quinolone signal (PQS). PQS is a global regulator that governs the expression of RhlR-dependent virulence genes. The transcriptional regulator RhlR, as well as many RhlR-dependent genes, were found to be downregulated upon *FDH1*^Cb^ overexpression. As a result, we found an increased production of pyocyanin in the *nox*^Sp^ strain. Pyocyanin is a blue redox-active secondary metabolite and an important RhlR-dependent virulence factor involved in the progression of infection and biofilm formation (30) (Fig. S5).

10.1128/mBio.02434-21.7 FIG S5 Pyocyanin content in *P. aeruginosa* overexpressing *nox*^Sp^ and *FDH1*^Cb^. Download FIG S5, PDF file, 0.1 MB. Copyright © 2022 Arce-Rodríguez et al. 2022 Arce-Rodríguez et al. https://creativecommons.org/licenses/by/4.0/ This content is distributed under the terms of the Creative Commons Attribution 4.0 International license.

Since PqsH, which catalyzes the terminal step in PQS production, synthesizes PQS using the substrates 2-heptyl-4-quinolone (HHQ), NADH, and oxygen (31), it might be speculated that an increased NADH-linked oxidative phosphorylation leads to a lack of cofactors for the biosynthesis of PQS and thus contributes to the lower PQS levels in the *FDH1*^Cb^ strain and thus the low-virulence phenotype. Interestingly, PQS governs also the production of genes involved in oxidative stress defense such *ahpC*, *katA*, and *sodB* (32). Their reduced transcription in the *FDH1*^Cb^ strain (despite enhanced *oxyR* promoter activity) might thus also be explained by low PQS levels.

## DISCUSSION

In the last 10 years, our understanding of the bactericidal activity of antibiotics has undergone a paradigmatic shift. It has been widely accepted that death is directly caused by lesions specific to a particular class of antibiotics. However, bactericidal antibiotics also lead to the cellular accumulation of toxic reactive oxygen species, which in turn make an important contribution to cell death.

Several studies demonstrated that antioxidants and iron chelators, which interfere with the production of ROS, protect cells from antibiotic killing and thus supported the ROS-lethality hypothesis. In this study, we took a different approach, and as opposed to scavenging ROS, we manipulated cellular metabolism directly to produce an imbalance in cellular redox homeostasis. Our data indicated that the excess of NADH produced by the formate dehydrogenase in P. aeruginosa produces a concomitant increase in the membrane potential and a boost in the production of ROS, while the overexpression of the NADH-specific oxidase (Nox) led to a drain of NADH and to a decrease in the intracellular pH.

With this, the outcome generated by the activity of FDH1^Cb^ resembles that of antibiotic treatment. However, while antibiotic treatment induces a hyperrespiration condition presumably either directly via activation of the TCA cycle or as a consequence of an enhanced need for ATP, the enzymatically produced NADH burst seems to induce hyperrespiration but lacks the primary attack elicited by the antibiotic. The fact that the generation of ROS in our approach is independent from antibiotic treatment provides the unique opportunity to test whether shifts in redox balances sensitize the bacterial cell to antibiotic killing and thus whether the ROS-lethality hypothesis is supported.

We found that the excess of NADH, which is directly related to the formation of ROS in the *FDH1*^Cb^ strain, resulted in a more resistant phenotype against the fluoroquinolones and amikacin as determined by agar diffusion (Fig. 4) and growth inhibition assays (Fig. S3). Enhanced antibiotic efflux by P. aeruginosa RND pumps seemed to account for this phenomenon, as we observed an enhanced transcriptional expression of the efflux pumps in the *FDH1*^Cb^-overexpressing strain. Furthermore, mutational studies revealed that the activity of the MexAB and MexXY but not the MexCD and MexEF P. aeruginosa efflux pumps contributed to the higher resistance levels in the *FDH1*^Cb^-overexpressing strain. Both aminoglycosides and fluoroquinolones are known substrates of the MexAB and MexXY efflux pump (33, 34), whereas ceftazidime and meropenem, toward which the *FDH1*^Cb^-overexpressing strain did not develop resistance, are less good substrates.

Of note, resistance levels were not found to be elevated for tobramycin in the *FDH1*^Cb^-overexpressing strain. One possible explanation could be that the specificity of binding of the two aminoglycoside (amikacin and tobramycin) substrates for the efflux pump might differ. Amikacin might be more efficiently pumped out of the cell than tobramycin. Another explanation could be related to the increase in membrane potential observed in this strain. In hyperpolarized cells (as is the case with the *FDH1*^Cb^-expressing strain), the gradient of ions across the membrane generates a strong electrochemical potential that is used by the cells as a source of energy to transport molecules against a concentration gradient (35). This process (known as the ion motif force or IMF) is crucial for the uptake of positively charged antibiotics such as aminoglycosides (36). Thus, differences in the ionization constant (pK_a_) of the two aminoglycosides, tobramycin and amikacin (37), might account for the observed differences, as an increased *FDH1*^Cb^ dependent intracellular import of tobramycin might nullify the effect of *FDH1*^Cb^ expression on the enhanced efflux pump activity.

Our experimental setup reinforced the importance of efflux pump activity on resistance and revealed that higher MIC levels can be achieved by a higher activity of the pumps, which is promoted in the *FDH1*^Cb^-overexpressing strains. However, *FDH1*^Cb^ overexpression was associated with higher ROS levels, and thus the observed higher MIC values seem to stand in contrast to the ROS-lethality hypothesis. In this context, it is important to note that MIC values are determined under antibiotic exposure and assay growth inhibition, whereas killing, which is promoted by ROS, is measured as bacterial survival after stressor removal. The two processes, growth inhibition and bacterial killing, have been demonstrated to be mechanistically distinct, and there are mutations and chemicals that affect only killing or the MIC levels (38, 39). In this study, we found that the killing activity of ceftazidime and tobramycin significantly differed between strains that overexpress *nox*^Sp^ and those that overexpress *FDH1*^Cb^. In *nox*^Sp^ strains, which exhibited reduced NADH levels, killing was decreased, clearly supporting the ROS-lethality hypothesis. Of note, we were unable to firmly establish that killing is a nonantibiotic specific effect, as the differences in the killing activity between the *nox*^Sp^- and *FDH1*^Cb^-overexpressing strains for amikacin, levofloxacin, or ciprofloxacin were either very small or nonsignificant. However, in contrast to ceftazidime and tobramycin, killing by these three antibiotics was very fast at the concentrations used in this study (10- to 40-fold MIC values). Thus, killing might have been too effective to need further support by toxic ROS activity.

It is noteworthy that the *nox*^Sp^-overexpressing bacterial cells, which were more resistant to killing by the bactericidal antibiotics (at least by ceftazidime and tobramycin), were not only characterized by low ROS levels but also by decreased intracellular pH levels. It seems conceivable that conditions that lead to a decrease of cytoplasmic pH exert a protective effect against antibiotic killing, whereas reduction of intracellular protons promotes cell death (9). The finding that not only elevated ROS levels but also the intrinsically linked intracellular proton levels might determine effectiveness of killing by bactericidal antibiotics implies that cellular bioenergetics in a more general sense might play an underestimated role in bacterial survival and the establishment of enhanced fitness. In this context, it is extremely interesting that following disturbances in the redox potential, which is accompanied by a drainage of protons from the cell, the cellular transcriptome shifts to a state that reflects a low-virulence phenotype. This phenotype—among others—is characterized by a lower expression of genes involved in the production of the interbacterial signaling molecule PQS and the lower expression of downstream virulence genes, including the global regulator gene *rhlR*.

Our findings open the way for the generation of novel potent drug combinations. Although there is the risk of impaired antibiotic efficiency due to an induced expression of efflux pumps, the combined use of bactericidal antibiotics with agents that disturb cellular redox homeostasis could still significantly enhance antibiotic killing. With this, sensitization of bacteria to currently available antibiotics could lead to a much more effective therapy to resolve life-threatening, chronic persistent infections. In addition, more effective killing promises to reduce overall antibiotic consumption and to mitigate selection pressure for resistance. A consequent slowdown in resistance development would be an achievement that cannot be valued highly enough. Last but not least, this drug combination could also exert important antivirulence activities. This antivirulence activity would, however, not be an alternative to the inhibition of bacterial growth but would add to the bactericidal activity of common antibiotics and thus maximize our chances to successfully combat devastating P. aeruginosa infections.

## MATERIALS AND METHODS

### Bacterial strains, DNA techniques, and culture conditions.

The strains and plasmids used in this study are listed in Table S1. All P. aeruginosa derivatives were engineered from reference strain PA14 (40). E. coli DH5α (41) was used for the routine propagation and construction of plasmids. General methods for DNA manipulation were performed with standard protocols described elsewhere (42). The mobilization of plasmid DNA into E. coli was carried out by chemical transformation of cells prepared with a solution of rubidium chloride as described previously (43). For the case of P. aeruginosa, plasmids were incorporated by electroporation of cells washed and concentrated with 0.3 M sucrose as indicated by Choi et al. (44). Unless indicated otherwise, bacteria were cultured in LB medium (42) with agitation at 180 rpm at 37°C. 15 g L^−1^ Bacto agar (BD, Sparks, MD) was added as solidifying agent to prepare LB plates. For the determinations of intracellular pH, P. aeruginosa cells were grown in M9 minimal medium containing 8.5 g L^−1^ Na_2_HPO_4_⋅2H_2_O, 3 g L^−1^ KH_2_PO_4_, 1 g L^−1^ NH_4_Cl, 0.5 g L^−1^ NaCl, 0.5 g L^−1^ MgSO_4_⋅7H_2_O, 3.6 g L^−1^ (i.e., 20 mM) glucose, and 2.5 mL L^−1^ of trace elements solution (45). For the experiments involving the expression of C. boidinii’s formate dehygrogenase (*FDH1*^Cb^), 10 mM sodium formate were added to the culture media. Where necessary, media were supplemented with the following antibiotics to ensure plasmid maintenance: gentamicin (Gm) 10 μg mL^−1^ (for E. coli) or 50 μg mL^−1^ (for P. aeruginosa) or kanamycin (Km) 50 μg mL^−1^ (E. coli) or 500 μg mL^−1^ (P. aeruginosa). Unless stated otherwise, P. aeruginosa cultures were amended with the respective antibiotic only during the overnight precultures. Moreover, in order to avoid extra metabolic disturbances generated by the presence of these chemicals, the antimicrobials were drawn off from the culture by centrifugation (5 min; 8,000 × *g*), followed by two consecutive washes with the same medium. The washed cells were used to inoculate the respective cultures at the indicated optical density at 600 nm (OD_600_) in antibiotic-free medium. In addition, the MICs of the antibiotics meropenem (MEM), ceftazidime (CAZ), amikacin (AMK), tobramycin (TOB), ciprofloxacin (CIP), levofloxacin (LEV), and norfloxacin (NOR) were determined in P. aeruginosa carrying plasmid pSEVA634 (see below) using macrodilutions in LB medium as described previously (46).

10.1128/mBio.02434-21.1 TABLE S1 Bacterial strains and plasmids used in this study. Download Table S1, PDF file, 0.1 MB. Copyright © 2022 Arce-Rodríguez et al. 2022 Arce-Rodríguez et al. https://creativecommons.org/licenses/by/4.0/ This content is distributed under the terms of the Creative Commons Attribution 4.0 International license.

### Construction of NADH-controlling genetic devices.

In order to manipulate the intracellular concentrations of NADH, the coding sequence of C. boidinii’s formate dehydrogenase (*FDH1*^Cb^) and S. pneumoniae’s NADH oxidase (*nox*^Sp^) were cloned into plasmid pSEVA634 (47). The cargo module of this vector encodes the *lac*I^q^-P*_trc_* expression system, which is induced upon addition of IPTG. The *FDH1*^Cb^ gene was obtained as an EcoRI/PstI restriction fragment from plasmid pS234·*FDH1* (48), whereas the *nox*^Sp^ was digested from plasmid pS234·*nox* (49) using enzymes BamHI/PstI. Both genes were independently cloned into the same restriction sites of pSEVA634, resulting in the final pAAHFDH1 and pAAHnox1 plasmids.

### Growth curves.

To assay the growth of *FDH1*^Cb^- and *nox*^Sp^-carrying P. aeruginosa challenged with different classes of antibiotics, overnight precultures of the strains were grown in 2 mL of LB medium supplemented with Gm. As stated before, 1 mL of each preculture was harvested by centrifugation and washed twice with 1 mL of fresh LB. The cells were resuspended in fresh LB medium and diluted to a starting OD_600_ = 0.02 in LB supplemented with dilutions of each antibiotic and dilutions of the inducer IPTG (as indicated in each case). An aliquot of each sample (200 μl/well) was transferred into a Honeycomb 2 plate (Thermo Fisher Scientific, Waltham, MA), and the respective OD_600_ was recorded with an automated Bioscreen C MBR plate reader (Oy Growth Curves Ab, Helsinki, Finland).

### Preparation of cell extracts and *in vitro* enzymatic assays.

P. aeruginosa cultures were prepared in 20 mL of LB medium (supplemented with 10 mM sodium formate when necessary) at OD_600_ = ∼0.05 and induced with increasing concentrations of IPTG as indicated. The cells were grown to OD_600_ = 0.4 to 0.5 (∼1.5 to 2.5 h postinduction), harvested by centrifugation (15 min; 4,200 × *g*; 4°C), and resuspended in 3.2 mL of ice-cold 50 mM potassium phosphate buffer (pH 7.4) containing 100 mM 2-mercaptoethanol. Cellular contents were extracted by four pulses of sonication in a Branson Sonifier 250 (Branson Ultrasonics, Danbury, CT), followed by separation of protein extracts from the cell debris by centrifugation (30 min; 14,200 × rpm; 4°C). The total protein concentration of the extracts were determined by the Bradford method (50).

The enzymatic activities were assayed by monitoring spectrophotometrically the reduction of NAD^+^ (in the case of FDH1) or the oxidation of NADH (for Nox) at *λ* = 340 nm (*A*_340nm_). In the case of formate dehydrogenase, 90 μL of FDH buffer (50 mM potassium phosphate buffer [pH 7.4], 150 mM sodium formate, and 1.5 mM NAD^+^) were mixed with 10 μL of the cell extracts, and the *A*_340nm_ was automatically recorded every 30 s at 30°C in an EnSpire plate reader (PerkinElmer, Waltham, MA). For NADH oxidase, a similar method was performed with the following modification: the reaction mixture containing 98 μL of Nox buffer (50 mM potassium phosphate buffer [pH 7.4], 0.29 mM NADH, and 0.3 mM EDTA) was started with 2 μL of the cell extracts and incubated in the plate reader as indicated above. In both cases, the maximum linear rate obtained by plotting the *A*_340nm_ min^−1^, and an extinction coefficient (ε_NADH_) of 6.22 mM^−1 ^cm^−1^ was used to calculate the specific activity for each enzyme. In all cases, 1 unit of enzyme activity was defined as the amount of enzyme that catalyzed the formation/oxidation of 1 μmol NADH per min and per mg of total protein content.

### Quantification of intracellular pyridine nucleotides.

The intracellular NAD(P)^+^ and NAD(P)H were measured using a modification of the original *in vitro* cycling assay by Bernofsky and Swan (51) as described previously (52, 53). To this end, bacterial cultures were prepared from overnight precultures at an OD_600_ of ∼0.05 in 50 mL of LB medium (plus 10 mM sodium formate when necessary) and induced with 0.1 mM IPTG. The sample harvesting was carried out in cells growing in exponential (1.5 to 2 h postinduction; OD_600_ = 0.35 to 0.45), late exponential (2.5 to 3 h postinduction; OD_600_ = 1.0 to 1.2), and early stationary (4 to 4.5 h postinduction OD_600_ = 2.2 to 2.5) phases of growth. At each time point, 1.5 mL of the culture were harvested by fast centrifugation (1 min; 14,000 × rpm; 4°C), the supernatant was discarded, and the pellet was frozen by immersion in liquid N_2_. Cell pellets were stored at −80°C until further metabolite extraction. Additionally, 10 to 12 mL of the culture were aliquoted into preweighted 15-mL centrifuge tubes in order to calculate the cell dry weight (CDW). The cells were spun down (20 min; 4,200 × rpm; 4°C) and washed twice with 0.9% (wt/vol) NaCl, and after decanting the supernatant, the cell pellet was dried overnight at 80°C to a constant weight. The CDW was calculated by subtracting the weight of the preweighted 15-mL tubes to each sample.

For the extraction of the pyridine metabolites, the cell pellets were ice-thawed and suspended in 0.2 mL of either 0.25 M NaOH (for NAD(P)H extraction) or HCl (for NAD(P)^+^ extraction). Samples were heated for 15 min at 55°C, and the pH was neutralized by dropwise addition of 0.3 mL of either 0.1 M HCl (for NAD(P)H extraction)or NaOH (for NAD(P)^+^ extraction). Finally, the pH was equilibrated by the addition of 70 μL of 1 M bicine-NaOH buffer (pH 8.0), and the cellular debris was removed by centrifugation (5 min; 12,500 × *g*). A reaction mixture was prepared in a 96-well plate by mixing 15 μL of the supernatant with 80 μL of the reaction mixture (120 mM bicine·NaOH [pH 8.0], 0.5 mM 3-(4,5-dimethylthiazole-2-yl)-2,5-diphenyltetrazolium bromide [MTT], 4.5 mM EDTA, 4.5 mM phenazine ethosulfate), and the respective substrate for each reaction [either 200 mM ethanol for NAD(H/^+^) or 12.5 mM glucose-6-phosphate (G6P) for NADP(H/^+^)] determinations. The plates were incubated for 5 min at 30°C (avoiding light contact), and the reactions were started with the appropriate addition of 5 μL of either 350 U ml^−1^ of alcohol dehydrogenase or 5 U ml^−1^ of G6P dehydrogenase. The plate with the reaction mixtures was immediately placed into an EnSpire plate reader (PerkinElmer, Waltham, MA) prewarmed to 30°C, and the absorbance at 570 nm was monitored every 20 s. In parallel, standard curves were prepared by the addition of known amounts of either NAD^+^ or NADP^+^ to the reaction mixture and analyzed in the plate reader as described above. The concentration of nucleotides was calculated using the maximum linear rate of the absorbance at 570 nm (ΔA_570_ min^−1^) for the cell extracts and for the standard curves. The intracellular nucleotide content was finally obtained by dividing the concentrations by the CDW calculated previously.

### Measurements of membrane potential.

A *Bac*Light bacterial membrane potential kit (Thermo Fisher Scientific, Waltham, MA) was used according to the manufacturer’s instructions. In brief, overnight grown cells were inoculated in 10 mL of LB medium at OD_600_ = 0.05 and cultivated at 37°C until midexponential phase (OD_600_ = 0.4 to 0.5). IPTG was added to the cultures at 1 mM final concentration, and the samples were incubated again at 37°C. A small aliquot of each culture was taken before IPTG addition and 1 h (OD_600_ = 1.0 to 1.3), 2 h (OD_600_ = 1.5 to 2.2), 3 h (OD_600_ = 2 to 2.6), and 4 h (OD_600_ = 2.3 to 2.8) postinduction. The sampled cells were diluted in 0.5 mL of filtered PBS (0.22-nm pore size) to approximately 10^6^ cells ml^−1^ and exposed to 30 μM 3,3′-diethyloxacarbocyanine iodide [DiOC_2_(3)] prepared from a 3 mM stock solution in DMSO. After 30 min incubation in the absence of light, the stained bacteria were analyzed in an LSR Fortessa flow cytometer (BD Biosciences, San Jose, CA) equipped with a 488-nm laser excitation source and both green (525/50-nm bandpass filter) and red (685/35-nm bandpass filter) fluorescence emission channels. Forward and side scatter density plots were used to identify the bacterial cell population of interest and to exclude debris. Fluorescence was recorded for at least 20,000 bacteria in both green and red emission channels, and the red/green fluorescence ratios were subsequently calculated using population mean fluorescence intensities.

### Monitoring of ROS using a fluorescent reporter system.

To monitor the intracellular production of ROS, we resorted to measuring the transcriptional activation of *oxy*R (a widely known marker for oxidative stress). The promoter sequence of P. aeruginosa *oxy*R (PA14_70560) was obtained by PCR amplification of 300 bp upstream from the gene using primers aaPoxyR-F (5′-AAT TGA ATT CGA CAC TCC GGA GCC TGC AGA TC-3′) and aaPoxyR-R (5′-TAA TAA GCT TGG CTG CTC ATC CGT TAA GAT CG-3′). Both primers include EcoRI and HindIII restriction sites (underlined in each case), allowing the digestion and cloning of the P*_oxy_*_R_ PCR fragment into the same sites of the mCherry reporter vector pSEVA247R (47) and thus generating the plasmid pAAHPoxyR2 (Fig. 3C).

To measure the expression of the *P_oxy_*_R_-mCherry reporter, plasmid pAAHPoxyR2 was transformed into P. aeruginosa PA14 wild type (WT) or into the strains carrying the plasmid derivatives for expression of *FDH1*^Cb^ or *nox*^Sp^. These strains were grown overnight, and 1 mL of cells was centrifuged and washed to eliminate the antibiotics (see above). The cultures were started by diluting the cells to 0.005 OD_600_ in 10 mL of LB medium (plus 10 mM sodium formate, where necessary), followed by incubation at 37°C until midexponential phase (OD_600_ = 0.4 to 0.5). Either 1 mM IPTG (to induce the expression of *FDH1*^Cb^ and *nox*^Sp^) or different concentrations of ROS-generating compounds (Fig. S2) were added to the cultures and incubated again at 37°C. A small aliquot of each culture was taken before (untreated sample) and after 1, 2, and 3 h postaddition of the chemicals. The sampled cells were immediately diluted in 0.5 mL of filtered PBS (0.22 nm pore size) to approximately 10^6^ cells ml^−1^ and analyzed in the LSR Fortessa flow cytometer (BD Biosciences, San Jose, CA) equipped with a 561-nm laser excitation source and a 610-nm (20-nm bandpass filter) fluorescence emission channel. Forward and side scatter density plots were used to identify the bacterial cell population of interest and to exclude debris. The data were recorded for at least 20,000 bacteria, and the mCherry-specific signal was calculated using population mean fluorescence intensities.

### *In vivo* tracking of intracellular pH.

A method based on the ratiometric pH indicator protein PHP (54) was performed with the following modifications. The pH reporter plasmid pS2513·*PHP* was electroporated into P. aeruginosa previously carrying also the plasmids for expression of *FDH1*^Cb^ or *nox*^Sp^. Overnight precultures of these strains were grown in M9-glucose medium with Gm and Km to maintain the plasmids. Following, 1 mL of cells were centrifuged and washed as explained above to eliminate the antibiotics, and diluted to 0.05 OD_600_ in 10 mL of M9-glucose medium (plus 10 mM sodium formate, when necessary). The cultures were incubated at 37°C until the midexponential phase (OD_600_ = 0.3 to 0.4), and 1 mL of the cells was again centrifuged (5 min; 8,000 × rpm) and resuspended in 1 mL of fresh M9-glucose. Aliquots of 200 μL of each sample were distributed into 96‐well microtiter plates and incubated for 1 h at 37°C in an EnSpireplate reader (PerkinElmer, Waltham, MA) with orbital agitation at 180 rpm. After incubation, both the OD_600_ of the cell suspension and the fluorescence emission of PHP (λ_emission_ = 515 nm) at λ_excitation_ of either 405 or 485 nm were recorded every 2 min for 10 min. At this point, 2 μL of prewarmed (37°C) 0.1 M IPTG prepared in M9-glucose was quickly added to the wells, and the fluorescence recording was resumed for an additional hour.

In order to generate a calibration curve for the calculation of pH, another set of cultures containing 10 mL of M9 minimal medium were inoculated with P. aeruginosa harboring both pSEVA634 and pS2513·*PHP*. Carbonyl cyanide *m*‐chlorophenyl hydrazine (CCCP) was added to the cultures at 20 μM, and the cells were grown to the midexponential phase as detailed before. The subsequent cell sampling, collapse of cytoplasmic pH at different extracellular pH values, and calculation of the Boltzmann sigmoid best‐fitting calibration curve was performed as detailed by Arce-Rodríguez et al. (54). The intracellular pH at each time point was calculated from the ratio of fluorescence emission obtained at λ_excitation_ = 405 nm and λ_excitation_ = 485 nm plotted against a Boltzmann sigmoid calibration curve. The pH values are expressed as the mean value from at least three independent replicates ± standard deviation.

### Antibiotic susceptibility assays by the disk diffusion method and by Etest.

Overnight grown cells carrying either the EV or the *FDH1*^Cb^/*nox*^Sp^-harboring plasmids were centrifuged, washed, and diluted to approximately 10^6^ cells ml^−1^. 100 μL of cells were evenly spread onto 25 mL of LB-agar supplemented with 50 μg ml^−1^ Gm and with dilutions of IPTG (as indicated in each case). Either the antimicrobial disks (Oxoid, Basingstoke, UK) or an Etest stripe (bioMérieux, Marcy-l’Étoile, France) were placed onto the agar, and the plates were incubated at 37°C for 18 to 20 h or 26 to 30 h (in case of *FDH1*^Cb^- and *nox*^Sp^-expressing strains induced with 1 mM IPTG). Alternatively, blank disks were impregnated with 2 to 5 μL of the antimicrobial solution (according to the final required concentration) in the cases for which the antibiotic at issue was unavailable. For the disk diffusion assay, the diameter of the inhibition halo was measured for each antibiotic, and a ratio between the diameter generated by the EV control and the NADH-manipulated strains was calculated. In case of the Etest, the susceptibility to the different antibiotics was recorded following the manufacturer’s instructions.

### Antimicrobial time-killing assay.

Bacterial cultures were prepared from overnight grown cells at 0.05 OD_600_ in 20 mL of LB medium with 0.1 mM IPTG as inducer. The strains were cultured at 37°C until midexponential phase (OD_600_ = 0.4 to 0.6) and subsequently exposed to exceeding concentrations of the antimicrobial. One ml of the culture (taken before and at several time points after the antibiotic treatment) was spun down (5 min; 8,000 × rpm), and the cell pellet was resuspended in 1 mL LB. Appropriate dilutions of cells were plated onto LB-agar plates and incubated at 37°C, and the formation of CFU (CFU) was recorded every 24 h for a maximum of 3 days. A killing curve was plotted from the data assuming the CFU number obtained before antibiotic treatment as the 0 killing or 100% survival value.

### RNA isolation, sequencing, and downstream bioinformatics analysis.

Bacterial cultures (in biological duplicates) were prepared by diluting overnight-grown cells to OD_600_ 0.05 in 20 mL of LB medium, followed by incubation at 37°C until midexponential phase (OD_600_ = 0.4 to 0.5). At this point, the cultures were amended with 1 mM IPTG and incubated at 37°C in an orbital shaker. Samples for RNA isolation were collected in RNA protect (Qiagen, Hilden, Germany) after 1 h (in the case of the *nox*^Sp^ strain and its EV control) and 2 h (for *FDH1*^Cb^ strain and its control) postinduction and processed as described previously (55). rRNA was depleted using the RiboZero Gold kit (Illumina, San Diego, CA), and the cDNA libraries were generated with the ScriptSeq v2 kit (Illumina). The samples were sequenced as 50-nt single-end reads on an Illumina HiSeq 2500 instrument. Sequencing reads were mapped to the PA14 genome (NC_008463.1) with bowtie2 (56), and the reads per gene were extracted using featureCounts (57). Differential gene expression analysis between the strains harboring the empty vector and the bacteria expressing either of the enzymes was computed from the reads per gene using the *R* package edgeR (58). The obtained *P* values for differential expression were adjusted to account for the FDR using the method by Benjamini and Hochberg (59). Only genes with an adjusted *P* value of <0.05 and an absolute log_2_FoldChange > |1.5| were considered differentially expressed. The transcriptome sequencing (RNA-Seq) data are available at the Gene Expression Omnibus (GEO) under accession number GSE188801.

### Pyocyanin production.

The production of pyocyanin was quantified as described elsewhere (60). Briefly, midexponential growing cells (OD_600_ = 0.4 to 0.5) were induced with 1 mM IPTG as described in the previous sections. After 2 and 3 h induction, 1-mL samples were spun down (5 min; 13,000 × rpm), and 0.9 mL of the supernatant was mixed with an equal volume of chloroform by vigorous vortexing. The organic phase was recovered and mixed with 0.9 mL of 0.2 N HCl by vortexing. Aliquots of 0.2 mL from the aqueous phase were distributed into a 96-well plate, and the absorbance at 520 nm was measured in the EnSpire plate reader (PerkinElmer, Waltham, MA). Pyocyanin concentration (μg mL_supernatant_^−1^) was calculated by multiplication of the absorbance with a correction factor of 30.486 and divided by the cell density estimated at 600 nm.

### Statistical analysis.

Statistical significance between samples obtained from the enzymatic assays, quantification of pyridine nucleotides, measurements of membrane potential, and monitoring of ROS was calculated by unpaired *t* test with Welch’s correction. The differences between the inhibition zones obtained by the antimicrobial disk diffusion assays were assessed using ratio paired *t* test. All of these calculations were performed in GraphPad Prism v7.0 (GraphPad Software, San Diego, CA).

To evaluate the statistical difference in pH values between the samples, pH values from six independent experiments (recorded on two different days) were used. To compensate for variations dependent on the experiment day, values from each experiment day were ranked and were then merged again for each time point independently. *P* values were calculated using a Welch’s two-sample *t* test and the *P* values from the different time points were corrected using the false discovery rate (FDR). Only the comparison between the *nox*^Sp^ sample and the empty vector (EV) control resulted in FDR values of <0.05 at all calculated time points (Fig. S6). All calculations and analyses were performed in *R* (61). To check the reliability of the statistical approach, the *P* values were additionally calculated with randomized data sets. Even without *P* value adjustment, only 1.15% of all permutated (100,000 permutations) *P* values were below 0.05.

In order to determine the statistical differences between the time-killing curves, nonlinear regression models were fitted to the log_10_-transformed number of surviving CFU. In the case of TOB, AMK, LEV, and CIP treatments, killing parameters were fitted to a biphasic decay exponential model. For CAZ-treated cells, the killing curves were successfully fitted to the one-phase decay exponential model. In both cases, the data were fitted using least-squares (ordinary) fit (1.000 iterations), and the goodness-of-fit was checked by the adjusted *R*^2^ model. The best-fit killing rate constants over time (per hour) were compared statistically between each overexpressing strain and their respective empty vector control by means of an extra sum-of-squares *F* test. All the analyses were performed with GraphPad Prism version 7.0 (GraphPad Software, San Diego, CA).
