# Supplementary material for: Dual Effect: High NADH Levels Contribute to Efflux-Mediated Antibiotic Resistance but Drive Lethality Mediated by Reactive Oxygen Species
Source: mBio. 2022 Jan 18;13(1):e02434-21. doi: 10.1128/mbio.02434-21 (PMC8764520; doi:10.1128/mbio.02434-21)
Supplement: FIG S1 [file mbio.02434-21-sf001.pdf]

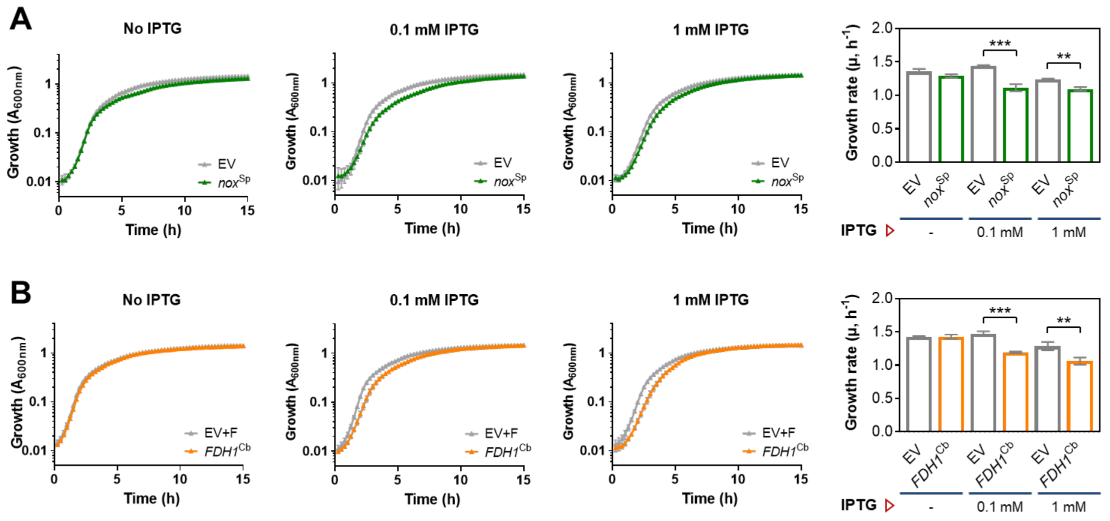

Fig. S1. Growth parameters of *P. aeruginosa* PA14 overexpressing (A) *nox*^{Sp} and (B) *FDH1*^{Cb} strains. Bacteria carrying the *nox*^{Sp} oxidase (A) or the *FDH1*^{Cb} (B) genes were grown in LB medium and the growth (absorbance at λ = 600 nm) was recorded in the *Bioscreen C* MBR^{TM} plate reader each 15 minutes. Cells carrying the empty vector (EV) served as a control and 10 mM sodium formate were supplemented to the cultures used for the *FDH1*^{Cb} strain and the respective vector control. Induction of the cultures was achieved by the addition of 0.1 mM or 1mM IPTG. The specific growth rate (µ) of the cultures (right panels) was calculated from the exponential growth by linear regression of log-transformed OD data. Statistical significance between µ values was calculated by means of a t-test (*p*-value: ** < 0.01; *** < 0.001).
