# Supplementary material for: Dual Effect: High NADH Levels Contribute to Efflux-Mediated Antibiotic Resistance but Drive Lethality Mediated by Reactive Oxygen Species
Source: mBio. 2022 Jan 18;13(1):e02434-21. doi: 10.1128/mbio.02434-21 (PMC8764520; doi:10.1128/mbio.02434-21)
Supplement: FIG S6 [file mbio.02434-21-sf006.pdf]

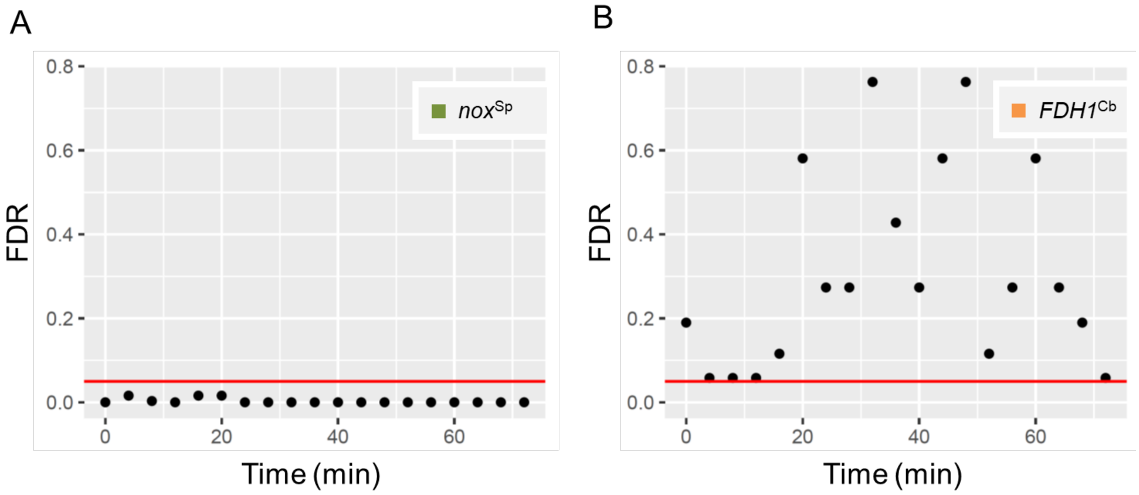

Fig. S6. Statistical differences of intracellular pH values. The differences between individual pH values obtained for the *nox*^{Sp} (A) or *FDH1*^{Cb} (B) strains and their respective empty vector (EV) controls were calculated using ranked data (separately for each experiment day) and a Welch's two sample t-test. Multiple testing for the different time points was corrected using the false discovery rate (FDR; check the main text for details). The final FDR calculated for each NAD^+/H manipulated strain vs EV is shown. The red line represents the limit FDR of 0.05. Calculations were performed at least in 6 individual replicates.
