# Supplementary material for: Dual Effect: High NADH Levels Contribute to Efflux-Mediated Antibiotic Resistance but Drive Lethality Mediated by Reactive Oxygen Species
Source: mBio. 2022 Jan 18;13(1):e02434-21. doi: 10.1128/mbio.02434-21 (PMC8764520; doi:10.1128/mbio.02434-21)
Supplement: FIG S2 [file mbio.02434-21-sf002.pdf]

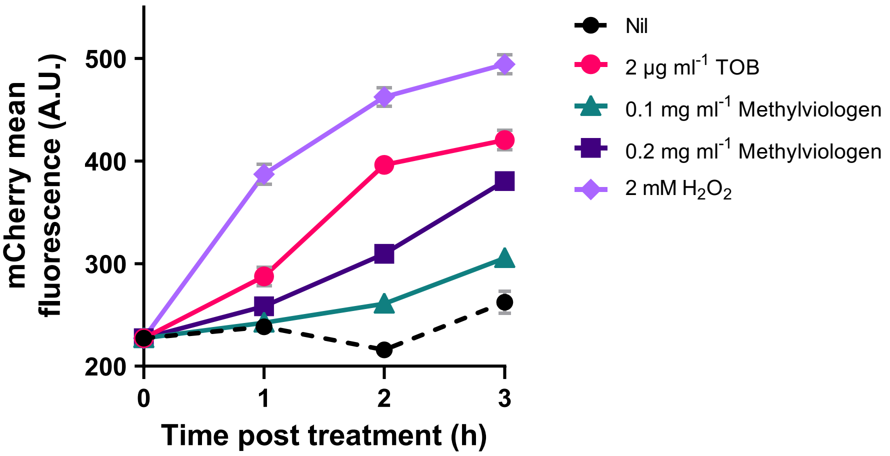

Fig. S2. Evaluation of the pAAHPoxyR2 as an intracellular ROS reporter. PA14 cells carrying the pAAHPoxyR2 vector-borne sensor were cultured in LB medium to early exponential phase of growth. At this point, widely known oxidative stress promoting compounds were added to the cultures and the specific mCherry signal was recorded each hour by flow cytometry. The plots show the mean fluorescence intensities of the samples calculated from the whole cell population. The experiment was carried out in triplicates. Statistical differences were assessed by means of an unpaired t-test with Welch's correction (*p*-value: ** < 0.01; *** < 0.001; **** < 0.0001).
