# Supplementary material for: Dual Effect: High NADH Levels Contribute to Efflux-Mediated Antibiotic Resistance but Drive Lethality Mediated by Reactive Oxygen Species
Source: mBio. 2022 Jan 18;13(1):e02434-21. doi: 10.1128/mbio.02434-21 (PMC8764520; doi:10.1128/mbio.02434-21)
Supplement: FIG S3 [file mbio.02434-21-sf003.pdf]

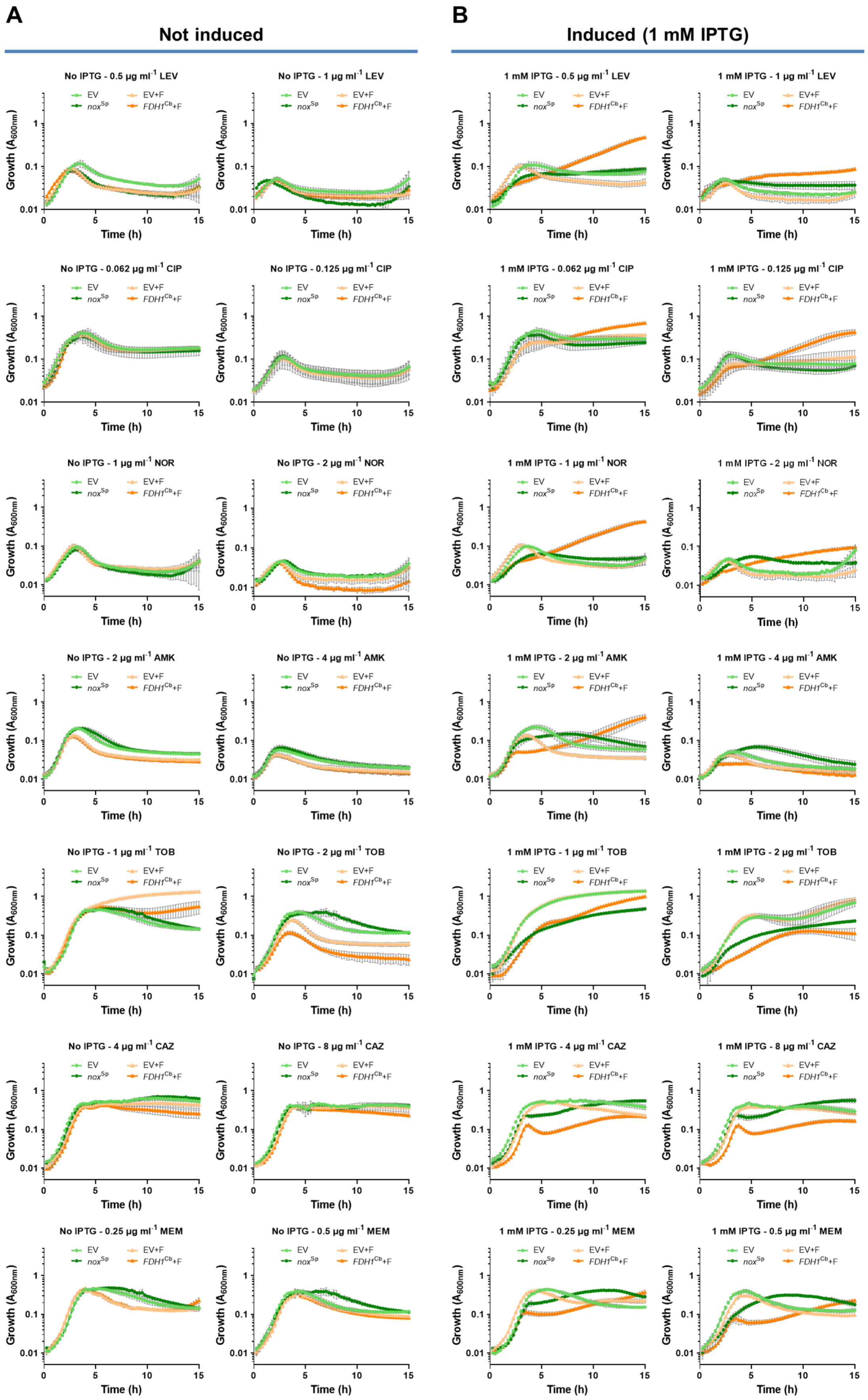

Fig. S3. Growth curves of *nox*^{Sp}- and *FDH1*^{Cb}-overexpressing *P. aeruginosa* PA14 challenged

with different classes of antibiotics. Bacteria carrying *nox*^{Sp} or the *FDH1*^{Cb} genes were grown in LB medium and the growth (absorbance at λ = 600 nm) was recorded each 15 minutes. Cells carrying the empty vector (EV) served as a control and 10 mM sodium formate were supplemented to the cultures used for the *FDH1*^{Cb} strain and the respective vector control. The cultures were started without (A) or with the addition of 1 mM IPTG (B), and supplemented with different concentration of antibiotics (specified in each case). The experiment was carried out in triplicates to ensure the reproducibility of the results. LEV: levofloxacin; CIP: ciprofloxacin; NOR: norfloxacin; AMK: amikacin; TOB: tobramycin; CAZ: ceftazidime; MEM: meropenem..
