# Supplementary material for: Dual Effect: High NADH Levels Contribute to Efflux-Mediated Antibiotic Resistance but Drive Lethality Mediated by Reactive Oxygen Species
Source: mBio. 2022 Jan 18;13(1):e02434-21. doi: 10.1128/mbio.02434-21 (PMC8764520; doi:10.1128/mbio.02434-21)
Supplement: FIG S4 [file mbio.02434-21-sf004.pdf]

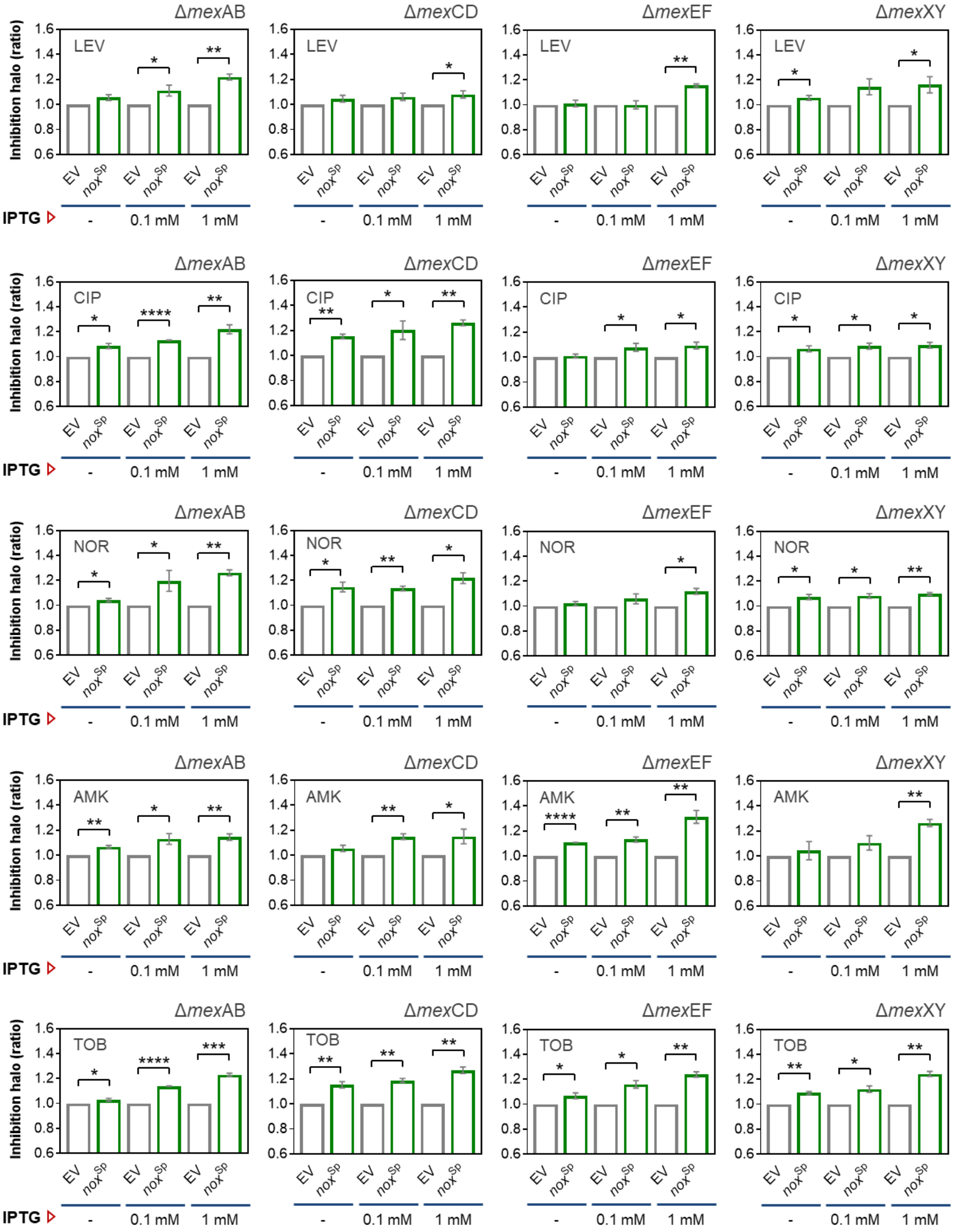

Fig. S4. Antimicrobial disk diffusion assay in *P. aeruginosa* efflux pump mutants overexpressing *nox*^{Sp}. Deficient strains in the main RND efflux pumps (Δ*mex*AB, Δ*mex*CD, Δ*mex*EF and Δ*mex*XY) expressing *nox*^{Sp} were plated onto LB-Agar with or without the inducer IPTG (0.1 mM or 1 mM, as indicated) and challenged with antibiotic-impregnated disks. Cells carrying the empty vector (EV) served as a control. The diameter of the inhibition zone of at least three independent experiments were measured and normalized to the empty vector. Significant differences between samples were calculated by means of a ratio paired t-test and are indicated by asterisks (*p*-value: * < 0.05; ** < 0.01; *** < 0.001; **** < 0.0001). LEV: levofloxacin; CIP: ciprofloxacin; NOR: norfloxacin; AMK: amikacin; TOB: tobramycin.
