# Supplementary material for: Dual Effect: High NADH Levels Contribute to Efflux-Mediated Antibiotic Resistance but Drive Lethality Mediated by Reactive Oxygen Species
Source: mBio. 2022 Jan 18;13(1):e02434-21. doi: 10.1128/mbio.02434-21 (PMC8764520; doi:10.1128/mbio.02434-21)
Supplement: FIG S5 [file mbio.02434-21-sf005.pdf]

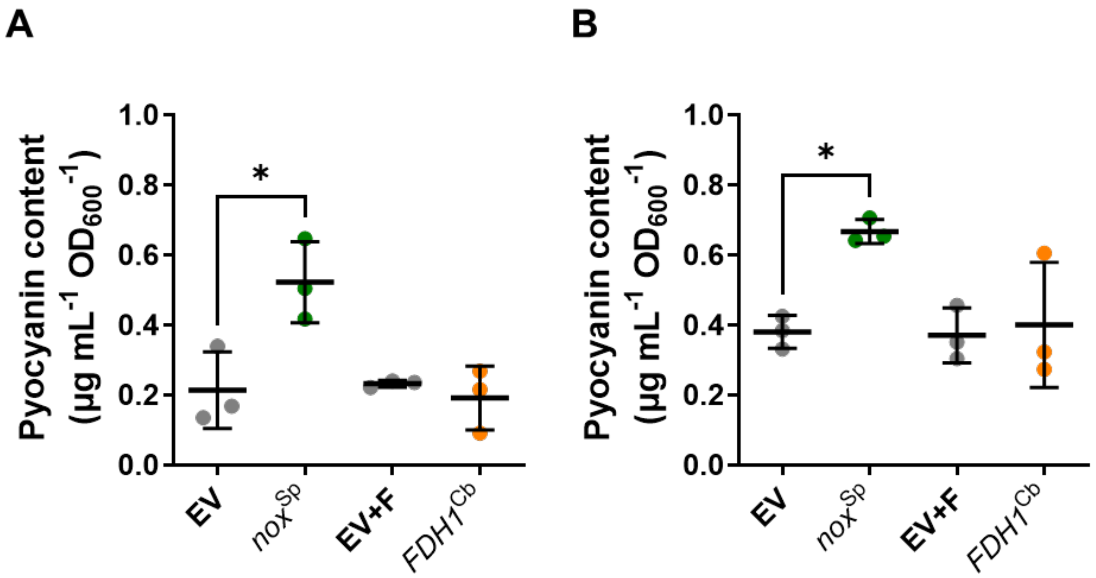

Fig. S5. Pyocyanin content in *P. aeruginosa* overexpressing *nox*^{Sp} and *FDH1*^{Cb}. Cells carrying the empty vector (EV) served as a control. Induction of the cultures was achieved in early exponential phase of growth by the addition of IPTG at 1mM. Samples were taken 2 h (A) and 3 h (B) post induction, pyocyanin was extracted in chloroform and quantified as detailed in the experimental procedures. The pyocyanin concentration of at least three independent experiments was calculated and reported as µg of the phenazine per ml relative to the OD_{600} of the culture. Significant differences between samples were calculated by the Mann-Whitney test (**p*-value < 0.05).
