# Supplementary material for: Dual Effect: High NADH Levels Contribute to Efflux-Mediated Antibiotic Resistance but Drive Lethality Mediated by Reactive Oxygen Species
Source: mBio. 2022 Jan 18;13(1):e02434-21. doi: 10.1128/mbio.02434-21 (PMC8764520; doi:10.1128/mbio.02434-21)
Supplement: TABLE S1 [file mbio.02434-21-st001.pdf]

Table S1. Bacterial strains and plasmids used in this study.

Strain/plasmid	Description / relevant characteristics	Reference
Strains		
Escherichia coli DH5α	F^-, *sup*E44, Δ*lac*U169, (φ80 *lac*ZDM15), *hsd*R17, (rk^- mk^+), *rec*A1, *end*A1, *thi*1, *gyr*A, *rel*A	(1)
Pseudomonas aeruginosa PA14	Wild type strain	(2)
P. aeruginosa Δ*mex*AB	PA14 strain with a complete deletion of *mex*AB genes	(3)
P. aeruginosa Δ*mex*CD	PA14 strain with a complete deletion of *mex*CD genes	(3)
P. aeruginosa Δ*mex*EF	PA14 strain with a complete deletion of *mex*EF genes	(3)
P. aeruginosa Δ*mex*XY	PA14 strain with a complete deletion of *mex*XY genes	(3)
Plasmids		
pSEVA634	Gm^R, *oriV* pBBR1, *oriT*, *lac*I^q/P_{trc} expression cassette	(4)
pS234·*FDH1*	Km^R, *oriV* pBBR1, *oriT*, *lac*I^q/P_{trc} controlling the expression of $FDH1^{Cb}$	(5)
pS234·*nox*	Km^R, *oriV* pBBR1, *oriT*, *lac*I^q/P_{trc} controlling the expression of nox^{Sp}	(6)
pAAHFDH1	GmR, pSEVA634 expressing the $FDH1^{Cb}$ under the control of *lac*I^q/P_{trc}	This Work
pAAHnox1	GmR, pSEVA634 expressing the nox^{Sp} under the control of *lac*I^q/P_{trc}	This Work
pSEVA247R	Km^R, *oriV* pRO1600/ColE1, *oriT*, promoterless vector including *mcherry* as a reporter gene	(4)
pAAHPoxyR2	Km^R, pSEVA247R cloned with the *oxy*R promoter. Used as a oxidative stress sensor	This Work
pS2513·*PHP*	KmR, *oriV* RSF1010, *oriT*, P_{EM7} → *PHP.* Used for the quantification of intracellular pH	(7)

REFERENCES

1. Hanahan D, Meselson M. 1983. Plasmid screening at high colony density. Methods Enzymol 100:333-42.
2. Friedman L, Kolter R. 2004. Genes involved in matrix formation in *Pseudomonas aeruginosa* PA14 biofilms. Mol Microbiol 51:675-90.
3. Seupt A, Schniederjans M, Tomasch J, Haussler S. 2020. Expression of the MexXY Aminoglycoside Efflux Pump and Presence of an Aminoglycoside-Modifying Enzyme in Clinical *Pseudomonas aeruginosa* Isolates Are Highly Correlated. Antimicrob Agents Chemother 65.
4. Silva-Rocha R, Martinez-Garcia E, Calles B, Chavarria M, Arce-Rodriguez A, de Las Heras A, Paez-Espino AD, Durante-Rodriguez G, Kim J, Nikel PI, Platero R, de Lorenzo V. 2013. The Standard European Vector Architecture (SEVA): a coherent platform for the analysis and deployment of complex prokaryotic phenotypes. Nucleic Acids Res 41:D666-75.
5. Akkaya O, Perez-Pantoja DR, Calles B, Nikel PI, de Lorenzo V. 2018. The Metabolic Redox Regime of *Pseudomonas putida* Tunes Its Evolvability toward Novel Xenobiotic Substrates. mBio 9.
6. Nikel PI, Perez-Pantoja D, de Lorenzo V. 2016. Pyridine nucleotide transhydrogenases enable redox balance of *Pseudomonas putida* during biodegradation of aromatic compounds. Environ Microbiol 18:3565-3582.
7. Arce-Rodriguez A, Volke DC, Bense S, Haussler S, Nikel PI. 2019. Non-invasive, ratiometric determination of intracellular pH in *Pseudomonas species* using a novel genetically encoded indicator. Microb Biotechnol 12:799-813.
